# Harnessing the structural determinants amenable for polypharmacological behavior of 7D against Sirt1 and CXCR3

**DOI:** 10.1016/j.isci.2026.116635

**Published:** 2026-06-29

**Authors:** Kiran Bharat Lokhande, Dhani Ram Mahato, Shailendra Asthana

**Affiliations:** 1 Computational Biophysics and CADD Group, Computational and Mathematical Biology Centre (CMBC), BRIC-Translational Health Science and Technology Institute, Faridabad, Haryana 121001, India

**Keywords:** Dual target, Sirt1, CXCR3, Benzimidazole, ePharmacophore, Computational Biophysics

## Abstract

Discovery of polypharmacological agents, inhibiting targets, either the same or diverse pathways, is an emerging paradigm in drug discovery. Designing such a candidate is challenging due to the lack of structural and dynamical consensus determinants of interacting partners. We identified a dual target inhibitor, 7D (mono-peptide of benzimidazole), a dengue antiviral lead against host proteins (Sirt1 and CXCR3) to underpin polypharmacological features. Targeting host proteins represents a compelling antiviral strategy as the modulation of host-regulated pathways can suppress dengue virus replication without directly engaging viral proteins, thereby minimizing the risk of resistance. Genesis of 7D discovery, critical determinants were identified for its rational designing to achieve Sirt1 selectivity. Focusing on the antiviral target, CXCR3 was explored via virtual screening of the in-house compound library. Among identified candidates, 7D appeared to be a potential hit, confirmed via *in vitro* and *in vivo* means, leading the basis to underscore the determinants responsible for polypharmacological behavior, paving a way for feature-based designing of such candidates.

## Highlights

• Elucidating structural determinants for polypharmacological features of 7D
• Common chemical scaffolds of 7D for its high affinity
• Conserved residues (with types) and water-mediated interaction networks are mapped
• 7D mimics the substrate binding pattern of P53 (in Sirt1) and PF4 (in CXCR3)

## Graphical abstract

**Figure:**
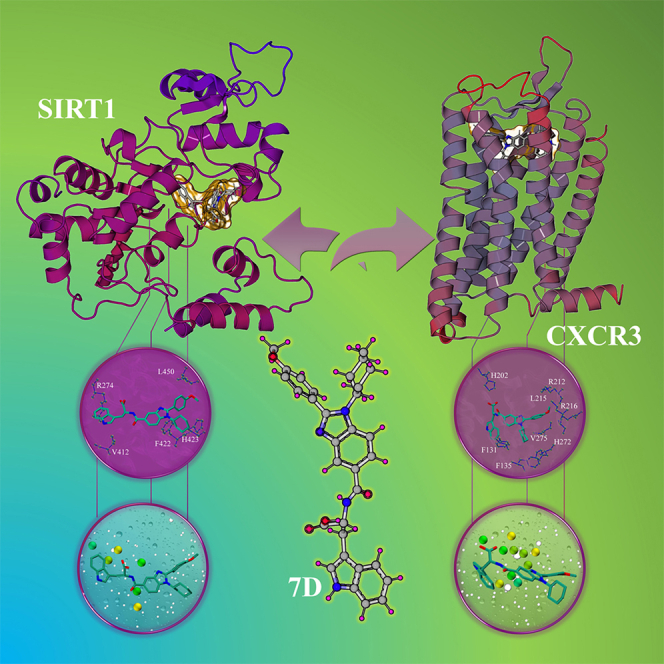

## Teaser

Properties of biomolecules; Molecular Structure; Bioinformatics

## Introduction

Clinical success of a drug is determined not only by targeting an individual protein, but also its interactions with many other proteins as well, which are also amenable to efficacy, safety, and toxicity. Identification of drug targets, either the same or diverse pathways whose inhibition can restore the physiological status of the cellular system from pathophysiology is desired; moreover, to inhibit such targets (more than one) via a polypharmacological approach is challenging. Identification of polypharmacological agent is an emerging paradigm in drug discovery that aims to develop drug candidates that modulate multiple molecular targets within a biological system as it can broaden the therapeutic scope with potentially greater efficacy and reduced risk of resistance.^1^ Capturing common features and parameters from interaction fingerprinting of both “drug” and “target” at molecular level are essential for designing polypharmacological agents as it is mostly serendipitous.

We have identified a potent anti-dengue candidate, 7D, a mono-peptide benzimidazole class which exhibits dual-targeting potential by inhibiting two targets, Sirt1 and CXCR3 of host machinery.^2^ Targeting host machinery offers a strategy to inhibit viral replication without directly engaging viral proteins, thereby minimizing the risk of resistance development.^3^^,^^4^

7D inhibition against Sirt1 was based on target selectivity basis,^5^^,^^6^ while against CXCR3 was identified from our *in-house* library (∼1.0 million compounds), which was virtually screened^2^ by mining the closest co-crystal. As a promising computational hit followed by *in vitro* and *in vivo* validation against both the targets, it is intriguing as how 7D is able to bind two distinct proteins? And is there consensus on molecular determinants that are responsible for this? With this quest, a detailed analysis of 7D is needed to be carried out against both targets to decipher the structural determinants such as types of interactions, their patterns, occupancy, binding site architecture in APO and HOLO forms, structural-dynamical changes, and possible mechanism of inhibition (substrate-dependent/independent) and so forth. to enlist them in a comparable manner.

While identifying the mechanistic basis of polypharmacological behavior of 7D, the determinants search begins with Sirtuins first, as 7D was rationally designed against it.^6^ Sirtuins (Sirt1-7) are nicotinamide adenine dinucleotide (NAD+)- dependent deacetylases that play critical roles in cellular metabolism, stress response, aging, and disease pathology.^7^^,^^8^^,^^9^ Among all Sirtuins, Sirt1 has been widely studied due to its regulatory role in transcriptional activity, epigenetic modifications, and metabolic homeostasis.^10^^,^^11^^,^^12^ Dysregulation of Sirt1 has been implicated in several pathological conditions, including viral infections.^13^ Despite its therapeutic potential, selective inhibition of Sirt1 remains a significant challenge due to a high degree of sequence and structural similarity among Sirtuin family members.^14^^,^^15^ Many structurally diverse small-molecule Sirt1-3 inhibitors have been reported.^16^^,^^17^^,^^18^^,^^19^^,^^20^ However, due to high amino acid conservation and structural similarity of the catalytic core among Sirt1-3, most of the reported inhibitors are either non-selective or of low micromolar potency. This further intensifies the need for the elucidation of the mechanism for selectivity between Sirt1-3 which was achieved in our previous work.^5^ We underscored the root cause of selectivity of Sirt1 among their homologs and based on structural and residual features, we designed selective Sirt1 inhibitor.^6^ The study conversed with the discovery of 7D that exhibited high selectivity for Sirt1 over Sirt2/Sirt3 with validated *in vitro* IC_50_ of 0.77 μM. Additionally, drug-likeness analysis confirmed that 7D possesses favorable properties and is non-toxic.^5^ This finding established 7D as a promising candidate to explore further in targeting Sirt1-related diseases.

Sirt1 is documented for its role in preventing viral growth.^21^ Building on the findings from our previous studies, we explored the broader biological effects of 7D, particularly its impact on dengue viral infections.^6^ Given the role of Sirt1 in regulating the immune response and viral replication, we investigated whether Sirt1 inhibition by 7D could enhance antiviral immunity. Our findings revealed that 7D-mediated Sirt1 inhibition led to increased interferon-stimulated gene (ISG) expression, improving host antiviral defense mechanisms.^2^ The study also demonstrated that 7D suppressed the viral replication of dengue virus (DENV) by modulating NF-κB and FOXO3a signaling pathways. Additionally, small-molecule inhibitors targeting Sirt1 have been shown to suppress the replication of both RNA and DNA viruses, as well as intracellular bacterial pathogens, in preclinical models.^22^ These results highlighted the potential of 7D in antiviral therapy.^6^

Another interesting aspect of Sirt1 is its N-terminal domain (NTD). The NTD residues are critical as they are responsible for its activation by Sirtuin-activating compounds (STACs) such as resveratrol.^23^ Full-length Sirt1 consists of three major regions: the NTD (183–229), the HDAC domain (229–516), and the C-terminal regulatory (CTR) segment. The NTD plays a key role in substrate recognition and STAC binding, contributing to the allosteric regulation of Sirt1. STAC binding to the NTD induces conformational changes that stabilize the active form of the enzyme and enhance catalytic activity.^24^ In our previous studies, this region (Gly183-Pro232) was not included. However, with the recent resolution of the Sirt1 structure (PDB IDs: 4ZZI,^18^ 4ZZJ,^18^ and 5BTR^25^) was used for understanding of NTD in 7D stability as its binding site, substrate binding site, and NTD binding zone is the same location.^18^ The inclusion of this region allows us for a more accurate representation of the full-length protein, thereby enhancing the reliability of our findings. The structural quality of all PDB entries used in this study was evaluated based on experimental resolution, sequence coverage, refinement statistics, and missing regions. Missing segments, where present, were modeled using prime. A summary of structural quality and preprocessing details is provided in Table S1.

While exploring the targets for antiviral discovery, chemokine receptors CXCR3 (G-protein-coupled receptor) have been explored as well, as they modulate excessive immune activation, moderate tissue damage, key regulators of immune cell trafficking and inflammatory responses, particularly in viral infections and autoimmune diseases.^2^^,^^26^^,^^27^ The CXCR3 mediates the migration of T cells and other immune cells in response to chemokines such as CXCL4, CXCL9, CXCL10, and CXCL11.^28^ Over activation of CXCR3 has been associated with autoimmune diseases,^29^ chronic inflammation,^30^ and viral infections,^31^ including dengue fever^32^ and COVID-19.^33^ Using a virtual screening approach, we have identified computational hits against CXCR3, in which 7D turn out to be the most active in experimental validations. We found that 7D effectively blocked CXCR3-mediated immune cell migration by disrupting the interaction between CXCR3 and its chemokine ligand, CXCL4. Previous studies confirmed that 7D binds to key residues within the CXCR3 orthosteric pocket, stabilizing the receptor in an inactive state, as confirmed via *in vitro* assays, as well as showing that 7D inhibited CXCR3-driven T cell chemotaxis and cytokine release, thereby preventing excessive immune activation and inflammation.^2^ Another *in vitro* assays have shown that 7D is able to inhibit all four dengue serotypes. Furthermore, inhibiting dengue infection in animal model AG129, confirming 7D as potential antiviral candidate against dengue.^2^ The *in vivo* data indicate the role of Sirt1 in regulating immune responses by suppressing excessive inflammation,^11^ while CXCR3 mediates immune cell recruitment to infection sites.^27^ Previous studies from our group demonstrated that Sirt1 inhibition enhances interferon production and improves viral clearance, whereas blocking CXCR3 prevents immune overactivation, reducing the risk of cytokine storms and tissue damage.^2^^,^^5^^,^^6^ Viral infections often exploit these pathways to evade host immune responses, and dysregulation of either Sirt1 and/or CXCR3 can contribute to disease progression. This relationship suggests that the dual inhibition of Sirt1 and CXCR3 could serve as a ***novel therapeutic strategy*** for managing viral infections, balancing immune activation and inflammation suppression.

Identifying small molecules that potentially inhibit the targets with corresponding cellular antiviral activity, confirming the experimental validation, and indicating the targets are viable, which reflects well as a considerable drop in dengue viral load was observed with the treatment of 7D. To address the underlying question that the antiviral effects of 7D are due to its binding at both targets, led us to a curiosity about the existence of common structural features that 7D utilizes to achieve polypharmacological behavior, or is it merely serendipity? Therefore, 7D’s binding behavior is characterized via mining the interactions in the binding and unbinding process, its type, physio-chemical properties, hydration, and energetic profiles. Since 7D bridges metabolic regulation (via Sirt1) with immune modulation (via CXCR3), it allows us to underscore target-specific common binding determinants for the discovery of polypharmacological candidates and their broad therapeutic application as antiviral. Overall, our earlier experimental studies demonstrated biological activity of 7D against Sirt1 and CXCR3; therefore, this work aims to investigate the structural and mechanistic basis underlying this experimentally observed dual-target behavior using an integrated computational framework.

## Results

### Cross-checking to confirm the most likely poses of 7D

Since the discovery of 7D and its binding pose was first reported against Sirt1,^5^^,^^6^ followed by CXCR3,^2^ to check the most optimized pose of 7D, BPMD was carried out. The BPMD analysis provides valuable insights into the binding stability of 7D with Sirt1 and CXCR3, focusing on the RMSD of the collective variable (CV) over time, which provides insights into the stability of the binding poses (Figure S1). The CV-based RMSD profiles track the deviation of the binding pose over time to obtain a comparative behavioral difference between both complexes. For Sirt1-7D, the RMSD starts at approximately 0.5 Å and steadily increases to around 2.6 Å over the simulation period. This progressive rise suggests moderate stability but also indicates the possibility that the ligand might undergo conformational shifts, deviating from its initial binding pose. In contrast, CXCR3-7D shows a slightly more stable profile, with RMSD starting at the same point but increasing more slowly and plateauing at around 2.0 Å. This plateau suggests that the ligand maintains a relatively stable conformation within the binding pocket of CXCR3 throughout the simulation.

BPMD pose evaluation is done based on persistence scores (PersScore) and PoseScore. The PersScore elucidates the binding dynamics, which is 0.45 in the case of Sirt1-7D, indicating that possibly key interactions are maintained, though with notable fluctuations during the simulation. Conversely, CXCR3-7D has a PersScore of 0.00, suggesting that no single interaction in terms of hydrogen bond persists consistently throughout the simulation. However, despite this, the lower CV RMSD observed for CXCR3-7D implies that the ligand remains stably bound within the pocket, possibly through dynamic rearrangements and transient but strong interactions. The Pose Scores complement these observations, with Sirt1-7D showing a higher PoseScore of 2.576, indicative of moderate pose stability but consistent with the increasing CV RMSD trend that reflects structural shifts over time. CXCR3-7D, on the other hand, has a lower PoseScore of 1.934, which, together with its lower CV RMSD, underscores the greater overall stability of its binding pose. This suggests that while individual interactions may fluctuate, the overall conformation of 7D within the CXCR3 binding pocket remains stable. This analysis confirms that 7D in both cases has shown stability, however slightly better in the case of CXCR3. In order to benchmark the binding affinity and pose stability of 7D against established modulators, additional docking and MM/GBSA analyses were performed for known inhibitors, including EX527 and Tenovin-1 for Sirt1, and AMG487 and Melatonin for CXCR3. As summarized in Table S2, 7D demonstrated stronger docking affinity toward Sirt1 and CXCR3 as compared to known inhibitors. The observed binding orientation of 7D also provides a structural rationale for the previously reported SAR trends among related analogs . In particular, the aromatic scaffold and hydrophobic substituents of 7D facilitate stable hydrophobic interactions within the Sirt1/CXCR3 binding pockets, whereas analogues lacking these interaction features previously exhibited comparatively reduced activity.^6^ Similarly, the positioning of hydrogen bond donor/acceptor groups in 7D enables favorable interactions with key residues identified in the docking and MD analyses, which may contribute to its experimentally observed dual-target activity profile.^5^

### Binding pose-mode characterization of 7D at Sirt1 with N-terminal

In post-processing analysis and characterizing the binding pocket, we underscored that the binding pose of 7D localizes at the substrate-binding site. Our previous work was N-terminal independent, as we had truncated the NTD to clearly understand how 7D is able to achieve a similar orientation as acetylated lysine (the natural substrate). Notably, 7D aligns itself in a comparable fashion, which was cross-validated through MD simulations. Since it has been reported and discussed that the NTD of Sirt1 plays a crucial role in substrate recruitment, and the current binding pose of 7D aligns with this region, it became crucial to investigate the functional impact of the NTD. Furthermore, the slight increase of CV-RMSD in BPMD is due to the lack of N-terminal, as it is localized at the 7D binding site) which was not considered in previous work. Therefore, the N-terminal region was incorporated in this study to observe any conformational influence, either in terms of 7D orientation or changes in the binding site residue architecture. To capture this, we selected three available Sirt1 crystal structures that represent a continuum of N-terminal conformations, ranging from closed to open states: PDB IDs 5BTR (fully closed),^25^ 4ZZI (partially closed),^18^ and 4ZZJ (fully open).^18^ Incorporation of these crystals provides a diverse range of N-terminal orientations. 7D was docked into all three structures and, *a priori*, it was able to occupy the same substrate-binding site in each case, although with differences in its orientation from previously reported pose in case of 4ZZI and 4ZZJ, however, overlapped well in case of fully closed N-terminal structure (5BTR), indicating the possibility that N-terminal residues either not or slightly contribute in achieving this pose of 7D. Moreover, the BPMD analysis showed that the pose obtained in 5BTR has shown the best pose score among all other poses of 7D, indicating that the N-terminal possibly plays some role in better packing or stabilization of 7D, which is one of the fundamental questions to address in this work.

Furthermore, in the case of 4ZZI (partially closed NTD), 7D shifted from its initial position and moved slightly toward the NTD (tilted), though it remained aligned along the same axis as in the reported pose, indicating the potential for a sub-pocket or alternative yet overlapping binding pose. A similar outcome was observed with the fully open NTD conformation (4ZZJ) as well, further supporting the feasibility of this alternative pose. Since this new pose was found to be well-populated and energetically favorable, justifying its selection for further detailed analysis alongside the old pose.

**Figure 1:**
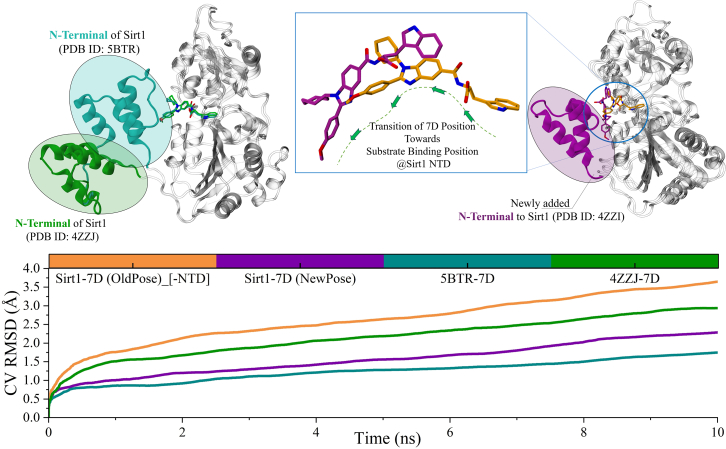
Pose optimization: a comparative BPMD analysis of 7D across different Sirt1 conformations The figure illustrates the conformational effect of the Sirt1 N-terminal domain on the binding dynamics of 7D. Sirt1 without the NTD (PDB ID: 4ZZI; truncated), representing the previously reported pose; Partially closed conformation with the NTD included (PDB ID: 4ZZI), where 7D shifts toward the substrate-binding site; Fully closed NTD conformation (PDB ID: 5BTR), showing stable 7D pose; Fully opened NTD conformation (PDB ID: 4ZZJ), where 7D exhibits increased mobility. See also Figure S1.

Comparative BPMD analysis across all four structural scenarios (Figure 1), revealed that the second tilted pose (identified in 4ZZI) exhibited enhanced stability, with a lower RMSD and higher persistence score compared to the old pose. Meanwhile, the fully open NTD conformation (4ZZJ) showed reduced pose persistence and higher flexibility, whereas the fully closed state (5BTR) supported a stable, restrained binding. These findings collectively highlight the overlapped binding pose (new pose) of 7D, in which the N-terminal possibly contributes. This data underscores the role of NTD in tuning binding pose diversity and supports the potential of 7D as a flexible ligand capable of adapting to multiple functional conformations based on conformational change of binding architecture.

### Structural dynamics of complexes Sirt1-7D vs. CXCR3-7D to capture the functional conformations

**Figure 2:**
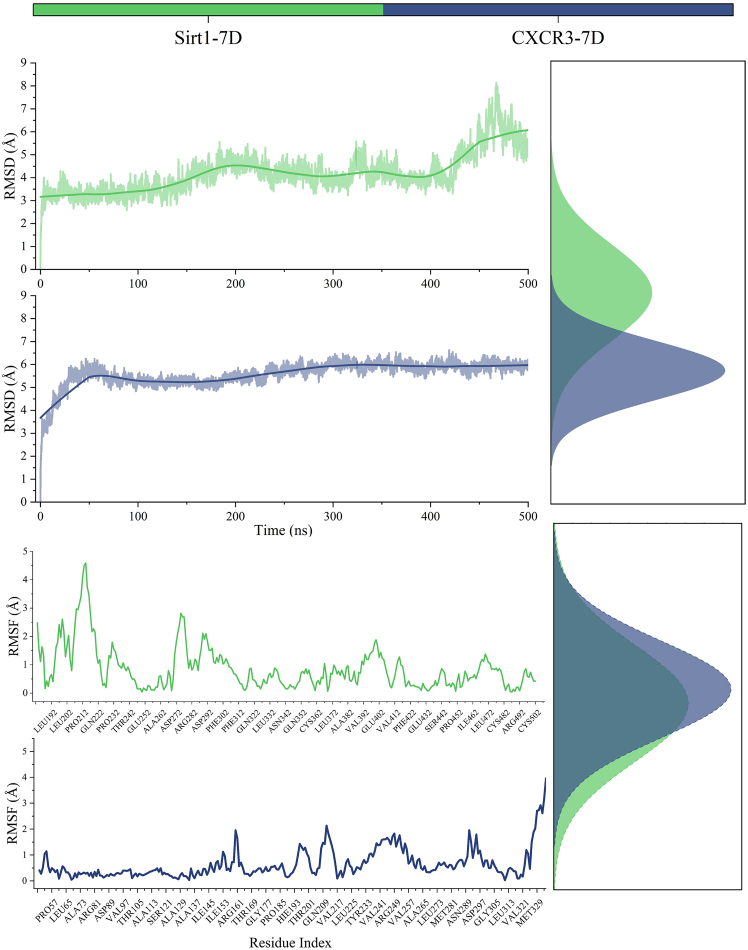
Comparative RMSD and RMSF Analysis of Sirt1-7D and CXCR3-7D complexes The top panel represents the RMSD plot illustrating the structural stability. The solid lines represent the smoothed trend, while the right-side panel shows the RMSD distribution, highlighting the overall structural deviations. The middle and bottom panels display the RMSF in Sirt1-7D (green) and CXCR3-7D (blue), respectively. The RMSF distribution on the right provides an overview of residue-wise flexibility. Higher fluctuations in the N-terminal region of Sirt1 suggest localized instability, whereas CXCR3 exhibits more stable and moderate fluctuations.

The RMSD and RMSF analysis illustrated in Figure 2 provides insights into the overall stability of the Sirt1-7D and CXCR3-7D complexes. The average RMSD for Sirt1-7D (4.18 Å) is lower than that of CXCR3-7D (5.58 Å), indicating that the Sirt1 complex maintains a more stable structure throughout the simulation. However, the standard deviation (SD) for Sirt1-7D (0.86 Å) is higher compared to CXCR3-7D (0.52 Å), suggesting that while the global structural deviation remains moderate, certain localized regions within the Sirt1 complex exhibit significant fluctuations, possibly in the binding site. In contrast, CXCR3-7D displays a higher average RMSD but with a narrower spread, signifying more widespread but uniform conformational changes rather than abrupt fluctuations. The RMSD distribution curves reinforce this observation, showing that Sirt1-7D experiences periodic structural variations, whereas CXCR3-7D undergoes consistent (Figure 2).

The RMSF analysis further supports these findings by highlighting residue-level flexibility. The Sirt1-7D complex shows prominent fluctuations in its N-terminal region, which may be attributed to flexible loop regions. This higher mobility at the N-terminus suggests that Sirt1 may undergo localized conformational rearrangements upon ligand binding. In contrast, CXCR3-7D exhibits a more evenly distributed fluctuation profile, indicating a global but controlled adaptation of the structure over the simulation period. The lower fluctuation levels in CXCR3-7D suggest that the complex maintains a more stable conformation across most of its residues, despite undergoing overall structural deviations as seen in the RMSD plot. It is evident that Sirt1-7D retains its core stability better but experiences localized flexibility, particularly at the N-terminal region. This may play a role in ligand recognition, packing, and interaction. On the other hand, CXCR3-7D undergoes larger conformational shifts but remains structurally uniform in its flexibility pattern, suggesting that the structural adaptation is more evenly distributed rather than confined to specific regions.

### ePharmacophore analysis of 7D against known inhibitors of both targets

We have extended the e-pharmacophore analysis by including standard compounds such as EX527, EX527∗, Resveratrol, EX243, AMG487, and MRV across multiple targets (Sirt1-3, CXCR3, and CCR5) (Figure S2). These compounds serve as benchmarks to compare 7D’s pharmacophoric profile and binding interactions. The analysis highlights the diversity and energy contributions of various pharmacophoric features, such as aromatic rings (R), hydrogen bond donors (D), and acceptors (A), which are crucial for ligand-target stabilization (Table 1).

**Table 1:** ePharmacophoric features of 7D and other standard compounds when complexed with their scores

Complex name	Pharmacophoric features with their score (kcal/mol)																
	A1	A2	D3	D4	D5	D6^a^	D7	H5	H6	H11	R8	R9	R11	R12	R13^a^	R14^a^	R15^a^
Sirt1-7D	–	–	–	–	–	**−1.05**^a^	−0.33	–	–	–	–	–	–	–	**−1.05**^a^	**−1.72**^a^	**−0.80**^a^
CXCR3-7D	–	–	–	–	–	**−1.60**^a^	–	–	–	–	–	–	–	–	**−0.85**^a^	**−1.12**^a^	**−0.72**^a^
Sirt1-EX527∗	−0.79	–	−0.33	–	–	–	–	–	–	–	−1.30	–	–	–	–	–	–
Sirt1-Resveratrol	–	–	–	−0.63	−0.32	**−0.32**^a^	–	–	–	–	−1.40	−0.69	–	–	–	–	–
Sirt2-Ex243	−0.66	–	−0.33	−0.83	–	–	–	−0.25	−0.58	–	−1.32	–	–	–	–	–	–
Sirt2-EX527∗	−0.66	–	−0.33	−0.86	–	–	–	–	–	–	−1.27	–	–	–	–	–	–
Sirt3-EX527	−0.49	–	−0.33	−0.23	–	–	–	–	–	–	-1L36	–	–	–	–	–	–
CXCR3-AMG487	–	−0.70	–	–	–	–	–	–	–	–	–	–	−1.27	−0.74	–	–	**−0.68**^a^
CCR5-MRV	–	–	–	−0.26	–	–	–	–	–	−0.30	–	–	–	–	**−0.33**^a^	**−1.80**^a^	–

Common pharmacophoric features shared between 7D and the respective standard ligand within the corresponding target binding site.

a Common features and their scores.

In the Sirt1-EX527∗ complex, significant features include a hydrogen bond donor (D3) contributing −0.33 kcal/mol and an aromatic ring (R9) with −1.30 kcal/mol. These interactions suggest that EX527∗ stabilizes Sirt1 via selective hydrogen bonding and aromatic stacking interactions. Similarly, in the Sirt1-Resveratrol complex, features such as hydrogen bond donors (D4, D5) with −0.63 and −0.32 kcal/mol, respectively, and an aromatic ring (R9) with −1.40 kcal/mol underscore Resveratrol’s stabilizing potential within the Sirt1 binding pocket. Notably, ligand 7D exhibits comparable interaction energies for key pharmacophoric features such as aromatic rings (R14: −1.72 kcal/mol) and hydrogen bond donors (D6: −1.05 kcal/mol), demonstrating its competitive binding affinity for Sirt1.

The analysis of Sirt2-EX243 and Sirt2-EX527∗ reveals interactions primarily mediated by hydrogen bond donors (D3, D4) and aromatic rings (R9), with energy contributions ranging from −0.66 to −1.32 kcal/mol. These values indicate stable ligand engagement within the Sirt2 active site. However, 7D’s pharmacophoric features for Sirt1 and CXCR3, particularly the strong aromatic ring interactions and hydrogen bonding, highlight its ability to achieve multi-target stabilization. In the Sirt3-EX527 complex, the pharmacophoric profile reveals interactions crucial for stabilizing the ligand within the active site of Sirt3. Key features include hydrogen bond donors (D4, D5) and aromatic rings (R8, R9), which contribute significantly to the binding energy. Notably, D4 and D5 exhibit interaction energies of −0.62 kcal/mol and −0.58 kcal/mol, respectively, indicating the presence of strong hydrogen bonds between the ligand and active-site residues. These interactions are complemented by aromatic rings R8 (−1.30 kcal/mol) and R9 (−1.22 kcal/mol), which contribute to the ligand’s overall stabilization via π-π stacking with aromatic residues in the binding pocket.

A comparison of 7D with EX527 demonstrates similar or stronger interactions in terms of hydrogen bonding and aromatic features. For instance, D6 and D7 in 7D display binding energies of −0.95 kcal/mol and −0.88 kcal/mol, respectively, which are higher than the hydrogen bond contributions of EX527 in the Sirt3 complex. Additionally, 7D’s aromatic rings (R14 and R15) exhibit robust binding energies of −1.72 kcal/mol and −1.12 kcal/mol, respectively, surpassing those of EX527. This suggests that 7D can establish stronger and more stable interactions within the Sirt3 binding site.

The similarities in the pharmacophoric features of 7D across Sirt1-3 highlight its ability to target multiple members of the Sirtuin family. These proteins share conserved catalytic domains, and the observed interaction profile of 7D with Sirt3 further supports its potential polypharmacological behavior. These observations may provide a basis for future exploration of multi-target modulation of Sirtuin-associated physiological processes, such as aging, metabolism, and stress response.

For CXCR3, the standard compound AMG487 shows a distinct pharmacophoric profile, with an acceptor (A2) contributing −0.70 kcal/mol and an aromatic ring (R11) at −1.27 kcal/mol. Although AMG487 provides robust binding interactions, 7D demonstrates comparable energy contributions, such as D6 at −1.60 kcal/mol and R14 at −1.12 kcal/mol, suggesting its capacity to stabilize CXCR3 effectively. Furthermore, the CCR5-MRV complex reveals strong binding contributions from R14 (−1.80 kcal/mol), indicating a preference for aromatic ring-mediated interactions. The shared features of 7D across Sirt1, CXCR3, and CCR5 reinforce its potential as a multi-target ligand.

**Figure 3:**
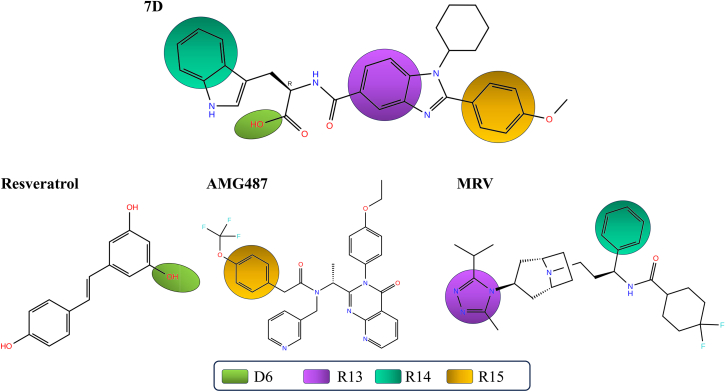
The figure illustrates representative functional motifs of 7D aligned with corresponding pharmacophoric elements present in established Sirt1 and CXCR3 modulators The comparison does not imply full structural similarity between the compounds; rather, it highlights shared functional regions relevant for target engagement. Color coding denotes analogous pharmacophore regions in 7D and the reference compounds: hydrogen bond-donor group (D6, green), central aromatic ring (R13, purple), aromatic ring (R14, teal), and distal hydrophobic tail region (R15, yellow). These highlighted features correspond to structural elements implicated in Sirt1 catalytic cleft recognition and CXCR3 transmembrane pocket engagement. See also Figure S2.

The comparative structural analysis of 7D with standard compounds, including Resveratrol, AMG487, and MRV, highlights the critical shared features that govern their binding against either Sirt1 or CXCR3 (Figure 3). These standard compounds serve as reference molecules due to their established interactions and biological activities.^5^ The structural overlap between 7D and reported inhibitors emphasizes common features such as functional groups in the head, a rigid core scaffold, and flexible tail regions, which collectively contribute to their efficacy as ligands. Resveratrol, a well-known activator of Sirt1, exhibits a hydroxyl-rich head group that forms strong hydrogen bonds with key catalytic residues, enhancing its binding affinity. Similarly, the head region of 7D features polar functional groups involved in hydrogen bonding, reflecting the interaction patterns of Resveratrol. This resemblance underscores the importance of the head region in anchoring ligands within the active site of Sirt1.

AMG487, an established known CXCR3 antagonist, shares an aromatic scaffold with the tail region of 7D, facilitating hydrophobic interactions and π-π stacking with binding site residues. This structural similarity ensures effective stabilization of the ligand-receptor complex. The tail of 7D, such as AMG487, provides the structural rigidity needed for proper orientation within the binding site, allowing optimal involvement of both the head and core regions. MRV, another standard compound, showcases a hydrophobic tail that extends into non-polar pockets of the receptor. This feature is reflected in the tail and core region of 7D. These groups not only enhance solubility but also interact with less conserved regions of the protein, improving the compound’s specificity and pharmacokinetic properties.

By combining features observed in Resveratrol, AMG487, and MRV, 7D incorporates functional elements from all three reference compounds (Table 1; Figure 3). The head region of 7D establishes strong interactions, similar to Resveratrol, ensuring specificity. The tail scaffold, similar to AMG487, facilitates hydrophobic interactions, stabilizing the ligand within the binding site. Finally, the core and tail regions are similar to MRV, contributing to flexibility, solubility, and selectivity. The detailed pharmacophore analysis, combined with experimental data, supports the claim of 7D as a polypharmacological agent. The e-pharmacophore models of 7D for Sirt1 and CXCR3 reveal overlapping features, including hydrogen bond donors (D6, D7) and aromatic rings (R13, R14, and R15), which are essential for stabilizing ligand-protein interactions. These shared features suggest that ligand 7D adopts a similar binding strategy across diverse targets, enhancing its potential to modulate multiple pathways effectively. The strong hydrogen bonding interaction of D6 (−1.60 kcal/mol) and the highly favorable aromatic ring interaction of R14 (−1.72 kcal/mol) in Sirt1 indicate robust binding, while similar features in CXCR3 highlight its dual-targeting capability.

### Capturing atomic features for complexes Sirt1:7D-vs.-CXCR3:7D

In the Sirt1-7D complex, five pharmacophoric features are identified: three aromatic rings (R13, R14, and R15) and two hydrogen bond donors (D6 and D7) (Figure S3A). The presence of multiple aromatic rings signifies regions where the ligand’s aromatic structures are involved in π-π stacking or hydrophobic interactions with the Sirt1 binding pocket. This alignment helps to anchor the ligand, making it energetically favorable within the binding site. The energy contributions of these ring features vary, with R14 exhibiting the lowest energy at −1.72 kcal/mol, which suggests a particularly strong interaction. This low energy value indicates a highly favorable interaction, likely contributing significantly to the ligand’s binding affinity for Sirt1. The R13 and R15 also provide stabilizing effects, with energies of −1.05 kcal/mol and −0.80 kcal/mol, respectively, adding to the complex’s overall stability. The hydrogen bond donor features, D6 and D7, represent core and tail areas, respectively, on the 7D capable of donating hydrogen bonds to complementary acceptor residues in the Sirt1 binding site. These donor features can form specific interactions, such as hydrogen bonds with polar or electronegative residues, further enhancing the ligand’s binding affinity. D6, with an energy contribution of −1.60 kcal/mol, indicates a substantial hydrogen bond donor interaction, suggesting it plays a critical role in the stabilization of the Sirt1-7D complex. D7, although less significant with an energy of −0.33 kcal/mol, still contributes positively to the complex’s stability.

In the case of CXCR3-7D complex features showing only four pharmacophoric elements: three aromatic rings (R13, R14, R15) and one hydrogen bond donor (D6) (Figure S3B). The aromatic rings in this complex also facilitate hydrophobic or π-π interactions with CXCR3. However, compared to the Sirt1-7D complex, these interactions are less energetically favorable, as reflected in the energy contributions. R14 has the strongest interaction at −1.12 kcal/mol, while R13 and R15 contribute −0.85 kcal/mol and −0.72 kcal/mol, respectively. This pattern suggests that the binding interactions within CXCR3 are weaker, potentially leading to a lower binding affinity compared to the Sirt1 complex. The single hydrogen bond donor feature, D6, with an energy of −1.60 kcal/mol, is comparable to the donor feature in the Sirt1-7D complex. This indicates that D6 may be forming a significant hydrogen bond with CXCR3, which is crucial for stabilizing the ligand in this complex. However, the lack of additional donor features in the CXCR3-7D complex limits the opportunities for specific polar interactions, potentially reducing the ligand’s overall binding stability in comparison to its interaction with Sirt1.

The Sirt1-7 complex shows a more diverse set of pharmacophoric features, including two hydrogen bond donors, which allow for multiple stabilizing interactions within the binding site. The distribution of both aromatic and donor features, along with their favorable energy contributions, indicates a strong binding profile. In contrast, the CXCR3-7D complex, with fewer features and lower interaction energies, may exhibit a less stable binding affinity. This analysis highlights the interaction patterns at the level of 7D within Sirt1 and CXCR3 binding environments based on the observed energetic and pharmacophoric features.

### 7D have features to bind on dual target as a polypharmacological agent?

Polypharmacology refers to the ability of a single molecule to interact with multiple targets, which can be particularly advantageous in addressing complex diseases where multiple pathways are involved. 7D demonstrated its activity against both Sirt1 and CXCR3 at experimental *in vitro* and *in vivo* level, indicating the possibility, while e-pharmacophore analysis also supports the possibility, as 7D’s activity against Sirt1 and CXCR3,^28^ seems to have properties that align well with the architecture of both targets, providing the basis to explore it as a polypharmacological agent. It is important to note that 7D was originally designed as a Sirt1-focused inhibitor based on benzimidazole-amino acid scaffold optimization. Its interaction with CXCR3 emerged serendipitously during subsequent biological evaluations, as reported previously.

In the hunt for common features, the alignment of the e-pharmacophore models for the Sirt1-7D and CXCR3-7D complexes revealed three common pharmacophoric features: D16 (hydrogen bond donor), R13, and R15 (aromatic rings) (Figure S3C). These shared features suggest that common interaction patterns may contribute to the observed engagement of 7D with both Sirt1 and CXCR3. The D16 donor feature likely forms stabilizing hydrogen bonds with conserved residues in the binding pockets of both Sirt1 and CXCR3, contributing to strong ligand-target interactions. Meanwhile, the R13 and R15 aromatic ring features may enhance binding stability through hydrophobic or π-π stacking interactions, which are crucial for proper ligand orientation and positioning within the active sites of both proteins.

The combination of experimental validation and shared pharmacophoric features supports 7D’s potential as a polypharmacological agent. By targeting both Sirt1 and CXCR3, ligand 7D could address multiple pathways, offering therapeutic advantages over single-target agents. This dual-target activity also offers an opportunity to optimize ligand 7D further for balanced affinity across both targets, potentially enhancing efficacy and reducing the need for combination therapies. To support the claim as a dual target inhibitor, a comparison is needed against documented inhibitors of both targets to capture the inhibitors' specific features.

The comparison with known inhibitors of Sirt1 and CXCR3 indicates that 7D's have common chemical features, which seem to have the property of a polypharmacological agent. The interaction energies associated with key pharmacophoric features are comparable to or exceed those of standard compounds, supporting the proposed dual-target interaction hypothesis. For instance, ligand 7D’s aromatic ring interactions in Sirt1 (−1.72 kcal/mol) and CXCR3 (−1.12 kcal/mol) surpass those of Resveratrol and AMG487, respectively. These structural observations are in strong agreement with our previously reported experimental findings,^2^ where 7D demonstrated dual modulation of the PF4-CXCR3 and P53-Sirt1 pathways, resulting in significant antiviral efficacy *in vitro* and *in vivo*. The current computational investigation provides mechanistic clarity to those biological results by revealing substrate-mimetic engagement within the Sirt1 catalytic cleft and prolonged stabilization within the hydrophobic transmembrane pocket of CXCR3. Together, the prior experimental validation and the present structural analysis provide a coherent and complementary framework explaining the polypharmacological behavior of 7D. While the dual-target host-directed strategy of 7D may reduce the likelihood of rapid viral escape mutations compared to direct-acting antivirals, such an approach does not inherently eliminate resistance-related risks. Host signaling pathways can undergo adaptive rewiring or compensatory regulation that may influence long-term therapeutic efficacy. Furthermore, modulation of host proteins such as Sirt1 and CXCR3 may carry context-dependent risks, including immune imbalance, pathway perturbation, or potential toxicity under certain physiological conditions. Since host-directed therapy has pros and cons both,^34^ therefore, careful therapeutic positioning and dosing strategies will be essential when advancing 7D as a host-targeted antiviral candidate.

#### Distance-wise multi-layered interaction fingerprinting of 7D

##### Sirt1 and CXCR3 binding site comparison

The interactions between 7D and the binding site residues in Sirt1 and CXCR3 were analyzed across three distance ranges from 2.5 Å, 3.5 Å, and 5.0 Å, respectively (Tables S3 and S4). The findings reveal distinct binding patterns, with certain similarities in residue types and binding regions across both complexes. At 2.5 Å distance, the ligand interacts primarily with hydrophobic residues in both systems. In the Sirt1-7D complex, residues such as Ala262 and Ile347 interact with the tail region of 7D, whereas in the CXCR3-7D complex, residues such as Leu106 and Ala113 exhibit similar interactions with the tail. This similarity in hydrophobic interactions highlights the role of these residues in stabilizing 7D within the binding pocket. At a 3.5 Å distance, both complexes feature interactions with charged residues. In Sirt1, Arg274 binds to the CT region of 7D, while in CXCR3, Arg216 interacts with the head region. Similarly, hydrophobic residues such as Pro207 (Sirt1) and Tyr60 (CXCR3) also contribute to ligand stabilization, demonstrating shared binding mechanisms across the complexes. At a 5.0 Å distance, diverse residue types, including polar, charged, and hydrophobic residues, contribute to the binding. For instance, residues Glu208, Ile411, and Ile316 in Sirt1 interact with the head, core, and tail regions of 7D, respectively. In CXCR3, residues such as Trp117, Gln204, and Leu190 interact with the core region, further strengthening the ligand’s binding affinity.

##### Overlapping residues and binding regions allows to underscore common determinants

**Figure 4:**
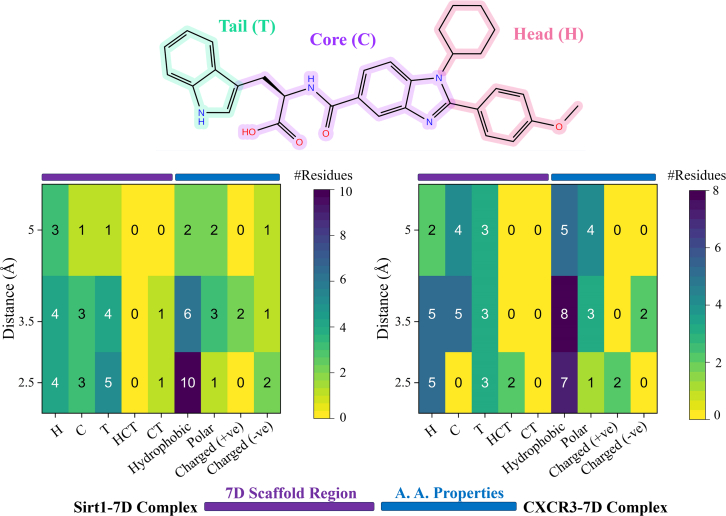
Comparative binding-site residue distribution and physicochemical properties surrounding 7D in Sirt1 and CXCR3 Number of amino acid residues categorized by regions of 7D head (H), core (C), tail (T), core and tail (CT), and head, core, and tail (HCT). Bars represent the distribution of residues in these regions for Sirt1 and CXCR3 at each distance. Distribution of amino acid types based on properties, viz., hydrophobic, polar, positively charged, and negatively charged residues, depending on their nature within the binding site region. Bars indicate the presence of these residue types for Sirt1 and CXCR3 at distances of 2.5 Å, 3.5 Å, and 5 Å. The data reveal overlaps in hydrophobic and polar residues and distinct patterns in charged residues and regional distribution. See also Table S4.

Residues with similar chemical properties and three-dimensional proximity to 7D were identified across both complexes. These overlaps suggest that the ligand 7D utilizes conserved binding features to interact with both Sirt1 and CXCR3. The charged residues play a key role in stabilizing the head region of 7D. Within 2.5 Å from the 7D binding position, residue Arg274 in Sirt1 and residues Arg212 and Arg216 in CXCR3 are interacting, indicating a conserved interaction motif for charged residues. Hydrophobic residues contribute significantly to 7D stabilization, particularly in the tail region. Another common set of interactions we noticed is that Leu418 in Sirt1 and Leu106 in CXCR3 are located within 2.5 Å of the 7D’s head and tail, respectively. Similarly, Phe273 in Sirt1 and Tyr271 in CXCR3 interact with the tail region within 2.5 Å, suggesting overlapping hydrophobic and aromatic interactions. Polar residues provide additional specificity to the interactions. Asn346 in Sirt1 and Asn132 in CXCR3 bind within 2.5 Å of the tail and head region of 7D, respectively. This similarity in polar interactions seems to contribute to 7D’s orientation and binding affinity in both complexes. The hydrophobic residues, including Ile316 (Sirt1) and Trp117 (CXCR3), stabilize the tail and core region of 7D within the binding pocket. Hydrophobic residues such as Ala262 (Sirt1) and Leu106 (CXCR3) dominate interactions in the tail region of 7D, ensuring 7D stability in both cases with similar types of residues (Figure 4).

The residue distribution in terms of distance around fragments (head, core, and tail) of 7D provides valuable insights into binding environments (Figure 4). Like in the head region, both proteins exhibit a significant number of residues at closer 2.5 Å and 3.5 Å distances. However, with respect to Sirt1, CXCR3 shows a consistent residue drop at 5.0 Å, indicating key residues are in the vicinity; however, at layer three (5.0 Å), residues are present in Sirt1 but not in CXCR3. For the core region of 7D, Sirt1 displays stable residue presence at 2.5 Å and 3.5 Å, but a reduced number of residues in 5.0 Å. Conversely, CXCR3 shows minimal interaction at 2.5 Å, while more presence of residues at 3.5 Å and 5.0 Å, suggesting differing interaction preferences for the core region between the two proteins. For the tail region, Sirt1 exhibits strong residue interactions at closer distances, which diminish significantly at 5.0 Å, whereas CXCR3 maintains consistent residue numbers across all distances, highlighting a difference in interaction dynamics in this region. Overall, this analysis indicates that tail is more compactly packed in CXCR3, while head is in Sirt1; however, core has shown an opposite trend of residue packing as it is high in Sirt1 and considerably low in CXCR3. These interactions fingerprinting along with the types of interactions at the binding site (hydrophobic, polar and ± charged residues) suggest that 7D utilizes overlapping binding mechanisms across Sirt1 and CXCR3, supporting its classification as a polypharmacological agent.

##### Dynamical stability measurement of most stable 7D pose

The initial pose of 7D in both cases was characterized in detail; furthermore, to check the dynamical stability, the MD simulations were carried out. In case of Sirt1, 7D starts moving from its initial old pose during MD simulation and stabilizes itself into another “new pose” which is an overlapped pose with “old pose,” in which it is shifted toward the NTD. Due to observing overlapped but different poses, this simulation was repeated in triplicate. The consistent appearance of a new pose indicates the possibility that more than one pose of 7D might exist in Sirt1. This change might be achieved due to the close packing of 7D and involvement of N-term using crystal structure 5BTR, which was truncated in the old pose. This new pose also shares key interacting features with its binding mode, suggesting that the alternative orientation of 7D also contributes well as it achieves better packing. while 7D pose in CXCR3 consistently retained its original binding position throughout the simulation, without evidence of an overlapping or secondary pocket. From the free energy landscape (FEL) analysis, it is evident that in the Sirt1-7D complex (Figure S4A), the initial binding pose of 7D lies in a distinct energy basin (start minima), which shifts during the simulation to settle into a different, deeper cluster of global minima. This supports the hypothesis that 7D relocates from its initial binding site and stabilizes near the NTD, indicating a dynamic repositioning toward a more favorable energetic conformation. In contrast, the CXCR3-7D complex (Figure S4B) displays two well-defined energy clusters. However, 7D maintains its initial binding position throughout the simulation, with no evidence of major conformational shifts. Among the ten lowest global minima identified, the deepest minima reside within the same cluster as the starting pose, confirming the energetic and structural stability of 7D in CXCR3. For further analysis, we selected the global minimum from this dominant cluster. To gain deeper insight, we characterized the key binding site features for both Sirt1 and CXCR3 at their respective energetically favorable (minima) poses by generating the FEL of most stable trajectories to capture the global minima state for both SIRT1 and CXCR3 bound to ligand 7D (Figure S4).

##### Minima state

**Figure 5:**
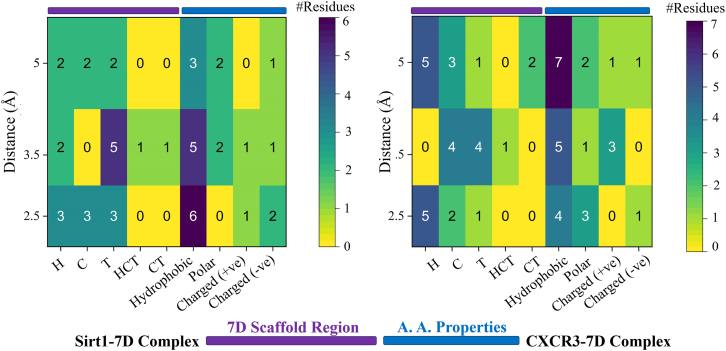
Residue distribution and their types around ligand 7D in the global minima structural binding sites of Sirt1 and CXCR3 See also Table S5.

From FEL, the residue distribution and interaction properties around 7D were analyzed specifically in this global minima state, providing deeper insights into the stable binding environment compared to the initial docked pose (Tables S5 and S6). This global minima conformation was further utilized for detailed interaction fingerprint analysis to understand key stabilizing interactions driving ligand binding. Figure 5 depict residual distribution and its properties around 7D at different distances. For the head region, Sirt1 exhibits a reduction in residue interactions at 2.5 Å and 3.5 Å, stabilizing at 5.0 Å, while CXCR3 shows a contrasting pattern with a complete absence of residues at 3.5 Å and consistent residue numbers at 2.5 Å and 5.0 Å. This suggests that in the global minima state, CXCR3 maintains stronger interactions at these distances in the head region of 7D compared to Sirt1, which exhibits a loss of interactions at closer proximities. The change in residue distribution highlights a shift in the dynamics of the head region interactions for both proteins.

In the core region, Sirt1 demonstrates a complete reduction in residues at 3.5 Å in the global minima state, with minimal changes at 2.5 Å and 5.0 Å, indicating weaker interactions compared to the initial state. On the other hand, CXCR3 shows a more balanced distribution in this region, with stronger residue presence at 3.5 Å, which slightly decreased at 5.0 Å. These observations suggest that the core region of 7D interacts more consistently with CXCR3 in the global minima state, while Sirt1’s interactions are significantly weakened, especially at intermediate distances.

The tail region of 7D shows contrasting behaviors for both proteins. Sirt1 exhibits stronger interactions at 3.5 Å, with residue numbers increasing compared to the initial state, but there is a slight reduction at 2.5 Å and 5.0 Å. In contrast, CXCR3 maintains a steady residue presence at 3.5 Å while interactions at 2.5 Å and 5.0 Å show variability. This indicates that the tail region of Sirt1 becomes more stable in the global minima state, while CXCR3 maintains consistent interaction dynamics across distances. Additionally, combined regions such as head-core (HC) and core-tail (CT) show minimal residue presence for both proteins, with only slight differences in distribution, reflecting limited interactions in these regions.

When examining the types of residues involved in interactions, both Sirt1 and CXCR3 display changes in hydrophobic, polar, and charged residue distribution. For hydrophobic residues, Sirt1 shows a decline in numbers at 2.5 Å and 3.5 Å but maintains similar interactions at 5 Å, whereas CXCR3 exhibits an increase in hydrophobic residues at 5 Å, supporting its preference for longer-distance hydrophobic interactions. Polar residues in Sirt1 are minimal at closer distances but increase at 3.5 Å and 5.0 Å, whereas CXCR3 shows a reverse trend with more polar residues at 2.5 Å in the global minima state. This highlights a difference in how polar residues contribute to ligand interactions in the two proteins.

Charged residues also exhibit distinct interaction patterns. Sirt1 retains negatively charged residues across all distances, showing stability in its charged interactions, while positively charged residues are observed only at 2.5 Å and 3.5 Å. In contrast, CXCR3 demonstrates a preference for positively charged residues at 2.5 Å and 3.5 Å, while negatively charged residues are minimal. This reflects a difference in the electrostatic environments of the two proteins around ligand 7D in their global minima states.

When comparing the global minima state to the initial docked pose, significant differences emerge in residue distribution and interaction preferences. Sirt1 shows a reduction in residue numbers in the head and core regions, with an increase in the tail region, suggesting a shift in interaction dynamics. CXCR3, on the other hand, exhibits more consistent interactions in the core and tail regions while losing residues in the head region at intermediate distances. Despite these differences, similarities in the presence of hydrophobic residues and the increasing prevalence of polar residues at longer distances are observed for both receptors.

Despite these differences, there are notable overlaps in interaction patterns that support the potential polypharmacological nature of 7D. Both receptors demonstrate a significant residue presence in the head region and strong hydrophobic interactions at closer distances, indicating shared binding preferences. Additionally, the increasing prevalence of polar residues at longer distances further highlights comparable residue environments in the vicinity of 7D for both Sirt1 and CXCR3. The similarities in residue distribution, hydrophobic interactions, and polar residue presence suggest that Sirt1 and CXCR3 share overlapping binding environments when interacting with the 7D.

#### Dissociation path interaction analysis unveil common determinants of 7D

The exit path interaction analysis of 7D from the Sirt1 and CXCR3 binding pockets reveals distinct patterns of molecular dissociation, providing insights into the stepwise loss of stabilizing interactions during forced unbinding, i.e., biased simulation. Given that ligand 7D is pulled by its tail, with its head initially embedded deep inside the binding cavity, the sequence of interactions observed across different frames highlights the key residues contributing to the molecular retention before the final dissociation event (Table S7).

**Figure 6:**
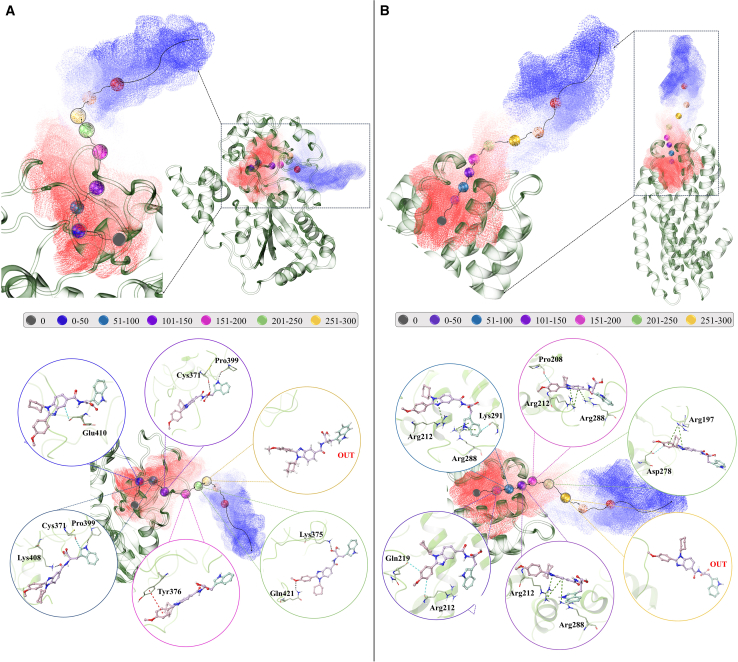
Differential dissociation mechanisms of 7D from Sirt1 and CXCR3 revealed by steered molecular dynamics simulations Steered molecular dynamics (SMD)-based dissociation pathway of 7D from (A) Sirt1 and (B) CXCR3. The figure depicts the forced unbinding (dissociation) trajectories of 7D obtained from SMD simulations and does not represent ligand association events. Panel (A) illustrates the sequential disruption of key stabilizing interactions within the Sirt1 catalytic cleft, showing transient retention of the benzimidazole head region prior to complete ligand release. Panel (B) demonstrates the progressive loss of interactions within the CXCR3 transmembrane binding pocket, with prolonged stabilization of the hydrophobic tail region before final dissociation. The observed interaction decay patterns and force-response profiles provide mechanistic insight into the differential unbinding behavior of 7D from the two targets. Association and equilibrium binding stability were evaluated separately using docking, IFD, equilibrium MD, and binding free-energy analyses described in earlier sections. See also Figures S10 and S11.

In the case of Sirt1, the initial interactions (frames 0–50) primarily involve the core region of 7D, with Glu410 forming hydrogen bond with the bond distance of 3.58 Å with aromatic residue, suggesting an early stabilization of the ligand within the pocket. As pulling progresses (frames 51–100), the tail region begins to dissociate, forming hydrogen bonds with Pro399 (1.83 Å) and aromatic hydrogen bond with Cys371 (3.49 Å), while Lys408 maintains a hydrogen bond (1.80 Å) with the core. Notably, in the 101–150 frame window, the Cys371 (2.12 Å) is now interacting with the core region of 7D (initially it was interacting with the tail), while the tail interacts with Pro399 by hydrogen bond (1.91 Å), marking the beginning of 7D’s exit. A significant shift occurs between frames 151–250, where the 7D’s head begins to interact with Tyr376 via pi-pi stacking (5.39 Å) and later with Gln421 (2.51 Å) and Lys375 (1.88 Å) by hydrogen bonds, indicating a final resistance before complete dissociation (Figure 6). This suggests that the head region of 7D is the last to unbind, demonstrating strong retention forces deep in the binding pocket. By frame 251–300, the 7D has fully exited, aligning with the observed energy trend (discussed in the later binding-free energies section) where Sirt1-7D undergoes a rapid energy increase during frames 150–300, reflecting the sharp loss of stabilizing interactions.

Whereas, the CXCR3 binding pocket exhibits a different exit pattern, with the head region involved in the stronger initial interactions (frames 0–50) compared to Sirt1. The aromatic hydrogen bonds with Arg212 (3.84 Å) and Gln219 (3.81 Å), suggesting a more anchored head positioning. As unbinding progresses (frames 51–100), Arg212 forms a pi-cation interaction (5.35 Å) with the core, while the tail region engages in dual pi-cation interactions with Arg288 (4.95 Å and 5.37 Å) and an aromatic hydrogen bond with Lys291 (3.10 Å). This indicates that CXCR3 retains the tail of ligand 7D longer than Sirt1 before complete unbinding, which may explain the gradual energy decline observed in the SMD energy profile. From frames 101–200, the core region maintains interactions with Arg212 and Arg288 via strong pi-cation bonding, while the head intermittently forms an aromatic hydrogen bond with Pro208 (3.79 Å). This suggests that 7D’s core remains stabilized within the binding site for a prolonged duration, preventing immediate unbinding. Interestingly, in frames 201–250, a strong interaction with Arg197 (pi-cation, 5.99 Å and 6.16 Å) and Asp278 (aromatic hydrogen bond, 3.33 Å) demonstrates a final resistance against unbinding, a distinct event from the Sirt1 pathway, where unbinding accelerates post-frame 150. Finally, at frames 251–300, 7D exits the CXCR3 pocket (Figure S5), aligning with the observed energy stabilization toward zero in the SMD energy analysis.

This exit pathway analysis suggests that CXCR3 retains 7D for a longer duration, evidenced by the sustained pi-cation and hydrogen bonding interactions in later frames (201-250^th^), whereas Sirt1 undergoes a sharper loss of interaction stability after frame 150. This aligns well with the SMD energy trends, where CXCR3-7D displayed a more controlled dissociation with prolonged resistance, while Sirt1-7D exhibited a faster energy surge, reflecting a more abrupt unbinding event. The prolonged interaction of 7D with CXCR3 suggests a stronger overall affinity and slower release, supporting the findings from MM-GBSA/MM-PBSA energy calculations, which indicated a higher binding free energy for CXCR3 compared to Sirt1.

#### Potential of mean force (PMF) analysis and dissociation energy calculation

The PMF plots generated from umbrella sampling provide a quantitative assessment of the FEL during the unbinding event of 7D from Sirt1 and CXCR3. For Sirt1-7D, the PMF plot (Figure S5A) illustrates a smooth, gradual increase in free energy along the reaction coordinate, reaching a final dissociation energy (Δ*G*) of −10.94 kcal/mol. This indicates a relatively moderate binding affinity, with a continuous energy barrier encountered as 7D moves away from the pocket. The energy landscape suggests that unbinding follows a stepwise process, where intermediate interactions slow down the escape of 7D, consistent with the exit path interaction data.

Whereas, for CXCR3-7D, the PMF plot (Figure S5B) reveals a higher dissociation energy of −13.50 kcal/mol, indicating a stronger binding affinity compared to Sirt1. The reaction coordinate shows multiple small energy barriers, which correlate with the persistent Pi-cation and hydrogen bonding interactions observed in the exit path analysis. The deeper free energy well in CXCR3 before final dissociation suggests a more stable binding mode, requiring additional energy to fully dissociate 7D, which aligns with the interaction analysis that highlighted prolonged retention of the ligand within the CXCR3 binding pocket.

These results provide an SMD comparison between Sirt1 and CXCR3, confirming that 7D exhibits stronger binding affinity for CXCR3 due to sustained electrostatic interactions, particularly involving charged residues such as Arg212 and Arg288. The PMF calculations validate the interaction analysis and highlight differences in ligand retention mechanisms across both proteins. The prior BPMD results also align with SMD and PMF analyses, confirming that compound 7D exhibits stronger and more stable binding with CXCR3 compared to Sirt1. The combination of lower RMSD and PoseScore for CXCR3-7D supports its higher binding affinity and stability.

#### Comparison of 7D binding-free energy contribution between conventional MD and accelerated SMD

The comparative binding energy analysis of 7D with Sirt1 and CXCR3 provides compelling evidence supporting its potential as a polypharmacological agent, interacting with considerable free energy with both Sirt1 and CXCR3 targets. The MM-GBSA binding free energy calculations indicate that 7D exhibits a significantly stronger interaction with CXCR3 (−56.20 kcal/mol) than Sirt1 (−37.92 kcal/mol). This trend is also observed with MM-PBSA calculations, where CXCR3-7D shows a binding energy of −46.37 kcal/mol, compared to −37.17 kcal/mol for Sirt1. These results suggest that 7D effectively binds to both targets, although with a stronger affinity toward CXCR3, strengthening its dual-targeting capability and its potential therapeutic relevance (Figure S6A).

Entropy calculations further demonstrate the polypharmacological nature of 7D by revealing that both complexes undergo notable conformational stability upon binding. The entropy value for CXCR3-7D is −27.36 kcal/mol, while for Sirt1-7D, it is −21.91 kcal/mol. The more negative entropy value for CXCR3-7D suggests that ligand 7D induces greater structural stability in CXCR3 compared to Sirt1. This behavior highlights 7D’s ability to adapt to different binding environments, a characteristic feature of polypharmacological agents capable of engaging both the targets with distinct structural properties.

Molecular dynamics and steered molecular dynamics simulations further emphasize the stability and interaction strength of 7D with both targets. The MD energy (ligand contribution energy) for CXCR3-7D (−22.24 kcal/mol) is notably more negative than for Sirt1-7D (−13.88 kcal/mol), indicating a more stable complex formation with CXCR3. Additionally, the SMD results demonstrate that CXCR3-7D requires −6.71 kcal/mol overall energy for the dissociation, whereas Sirt1-7D requires −4.57 kcal/mol. These findings indicate that ligand 7D remains tightly bound to both proteins under dynamic conditions, supporting its dual-targeting potential.

The free energy (ΔG) values, derived from MM-PBSA (enthalpy and entropy contributions), further indicate the polypharmacological nature of 7D. The calculated ΔG for CXCR3-7D is −19.01 kcal/mol, while for Sirt1-7D, it is −15.26 kcal/mol. These values incorporate both enthalpic and entropic contributions, confirming that 7D binds to both targets in a thermodynamically favorable manner. Despite the stronger binding to CXCR3, the substantial ΔG for Sirt1-7D confirms that 7D exhibits a meaningful interaction with Sirt1 as well, highlighting its potential as a dual-targeting inhibitor. This property is particularly relevant in drug discovery, where compounds capable of modulating multiple targets can provide enhanced therapeutic efficacy while reducing drug resistance.

These findings support the proposed dual-target interaction behavior of 7D toward both Sirt1 and CXCR3 within the evaluated computational framework. The favorable MM-GBSA and MM-PBSA binding free energies, the entropic contributions reflecting adaptability, the stable interaction profiles in MD simulations, and the resistance to dissociation in SMD all point toward the dual-targeting nature of 7D. The ΔG calculations further support the possibility of interaction of 7D with both targets and provide a mechanistic rationale for future investigation of dual-target modulation involving Sirt1 and CXCR3.

The SMD energy profile of 7D with Sirt1 and CXCR3 provides valuable insights into the dissociation process and the stability of these complexes under external force application. By analyzing the energy contributions across 500 frames in intervals of 50, we are able to trace how 7D dissociates from its binding pocket and evaluate its resistance to forced dissociation. The SMD energy data reveal a clear distinction in the energy dissipation pattern for both complexes (Figure S6B). In the initial frames (0–150), both complexes exhibit the strongest binding interactions, with CXCR3-7D showing significantly lower energy values (−19.38 to −13.02 kcal/mol) compared to Sirt1-7D (−5.82 to −15.00 kcal/mol). This suggests that ligand 7D forms a deeper and more stable interaction with CXCR3 in the early phase of dissociation, requiring greater force to disrupt these contacts. while Sirt1-7D reaches its maximum binding strength (−15.00 kcal/mol) within the 100–150 frame window, whereas CXCR3-7D follows a more gradual decrease in binding energy, indicating a stronger initial affinity and slower loss of interaction stability. As the dissociation progresses (frames 150–300), a significant energy drop is observed for both complexes, but at different rates. The energy for Sirt1-7D increases sharply from −15.00 kcal/mol to −0.84 kcal/mol, indicating a rapid weakening of interactions. Equally, CXCR3-7D displays a more controlled dissociation, with energy values transitioning from −13.02 kcal/mol to −1.89 kcal/mol, suggesting that ligand 7D retains its interactions with CXCR3 for a longer duration. This gradual release indicates a stronger overall affinity for CXCR3 compared to Sirt1, which aligns well with the MM-GBSA/MM-PBSA binding free energy results discussed earlier.

In the later frames (300–500), both complexes reach a minimal interaction state, where the energy values approach zero (no contact with protein, complete solvation). However, CXCR3-7D retains residual interactions (−0.024 kcal/mol), whereas Sirt1-7D completely loses its binding (−0.019 kcal/mol). This final stage confirms that ligand 7D dissociates more readily from Sirt1 than CXCR3. When comparing these findings with the MM-GBSA, MM-PBSA, and entropy results, a consistent trend emerges, supporting 7D’s dual-targeting capability. The higher binding free energies for CXCR3 (−56.20 kcal/mol in MM-GBSA and −46.37 kcal/mol in MM-PBSA) correspond well with the SMD energy results, where CXCR3-7D consistently shows stronger resistance to dissociation. Additionally, the entropy calculations (−27.36 kcal/mol for CXCR3 and -21.91 kcal/mol for Sirt1) further support the stronger conformational restriction induced by 7D in CXCR3, contributing to its prolonged binding stability.

#### Residual contribution via per-residue energy decomposition of complexes from stable (MD) and dissociation (SMD) pathways

**Figure 7:**
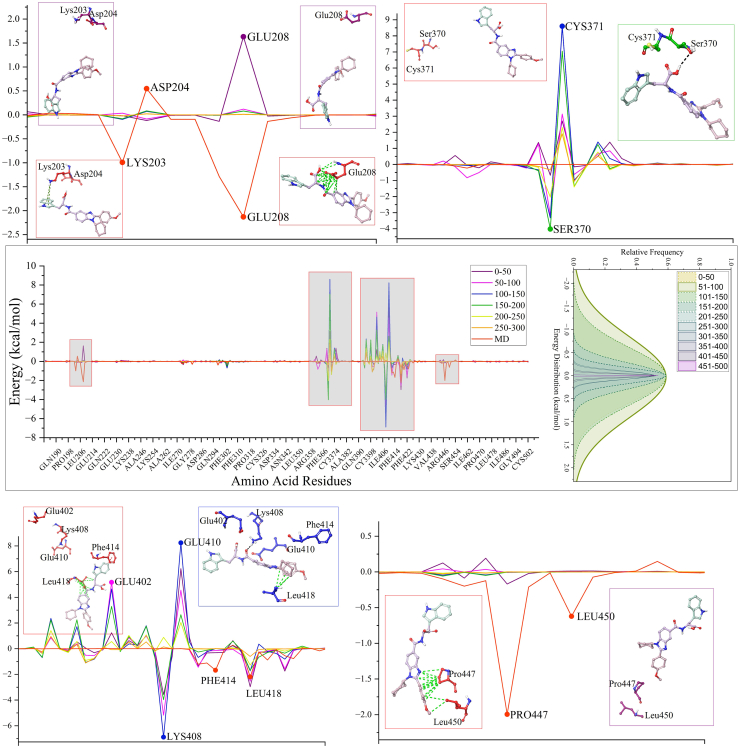
Per-residue energy decomposition profiles of the Sirt1-7D complexes under MD and SMD simulations across different frame intervals The plot highlights key residues contributing to binding stability and their interaction types, including H-bonds (black colored dashed lines), π-π stacking (red colored dashed lines), electrostatic interactions (dark green colored dashed lines), and good contacts (green colored dashed lines). The fluctuation of residue energies reflects the mechanical response of the complexes to applied forces during SMD.

**Figure 8:**
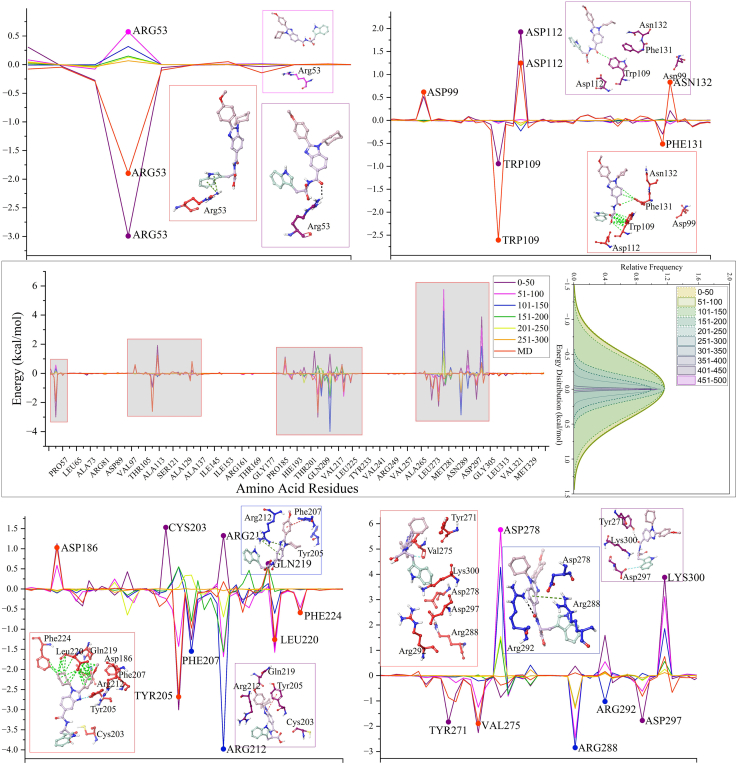
Per-residue energy decomposition profiles of the CXCR3-7D complexes under MD and SMD simulations across different frame intervals The plot highlights key residues contributing to binding stability and their interaction types, including H-bonds (black colored dashed lines), π-π stacking (red colored dashed lines), electrostatic interactions (dark green colored dashed lines), and good contacts (green colored dashed lines). The fluctuation of residue energies reflects the mechanical response of the complexes to applied forces during SMD.

The per-residue energy contributions of both the Sirt1-7D and CXCR3-7D complexes under MD and SMD conditions are illustrated in Figures 7 and 8, respectively, highlighting key residues involved in stabilizing interactions and their energetic fluctuations across different simulation intervals. In case of Sirt1-7D complex MD trajectory, residues such as Lys203, Glu208, Leu418, and Pro447 exhibit negative energy contributions, suggesting their roles in stabilizing the complex. Specifically, Glu208 shows a significant stabilizing energy of −2.13 kcal/mol, likely due to good contacts, while Leu418 contributes −2.19 kcal/mol, indicative of hydrophobic interactions. The presence of π-π interactions at Phe414 further supports the stability, with an energy contribution of −1.68 kcal/mol. As 7D transitions to the SMD trajectory, energy fluctuations become more pronounced. The residue Ser370 shows a sharp decline from −0.67 kcal/mol to −4.02 kcal/mol across the intervals, correlating with hydrogen bond formation that strengthens under mechanical stress. Similarly, Cys371 shifts from a stabilizing to a destabilizing role, moving from 2.71 kcal/mol to 8.60 kcal/mol, reflecting the disruption of contacts during stretching. Glu402 exhibits a similar trend, increasing from 1.24 kcal/mol to 5.17 kcal/mol, suggesting loss of favorable interactions as the complex undergoes deformation. Interestingly, Lys408 transitions from −3.57 kcal/mol to −6.88 kcal/mol, indicating that it forms stronger H-bonds in response to the mechanical forces applied. The energy decomposition for Leu418 maintains a stabilizing trend throughout SMD, consistent with its role in hydrophobic contacts that persist despite mechanical stress. This suggests that Leu418 contributes to the structural integrity of the complex even under dynamic conditions (Figure 7).

In case of CXCR3-7D MD simulations, Arg53, Trp109, and Tyr205 emerge as key stabilizing residues. Arg53 shows a strong stabilizing energy of −1.90 kcal/mol, attributed to π-cation interactions, while Trp109 contributes −2.61 kcal/mol, indicative of good hydrophobic contacts. Tyr205 provides a significant stabilizing force at −2.68 kcal/mol, likely from π-π stacking or H-bonds. During the SMD trajectory, energy decomposition highlights dynamic shifts. Arg53 initially becomes more stabilizing (−2.99 kcal/mol), reflecting strengthened H-bond interactions at the early SMD stages (0–50 frames). However, it destabilizes rapidly in later intervals, aligning with the loss of π-cation interactions as the complex is stretched. Asp112 transitions from a destabilizing to a slightly stabilizing role over time (1.93 kcal/mol to −0.23 kcal/mol), possibly reflecting transient electrostatic interactions. Tyr205 exhibits significant energy fluctuations, with a transition from −3.01 kcal/mol to positive values, indicating the progressive loss of its stabilizing influence, likely due to disrupted H-bonds and π-π stacking under mechanical force. Similarly, Trp109’s stabilizing contribution decreases from −0.94 kcal/mol to near-neutral values, indicating that its hydrophobic contacts weaken significantly under mechanical stress (Figure 8).

Residues such as Ser370 (Sirt1) and Arg53 (CXCR3) display enhanced stabilizing energies in early SMD stages, indicating stronger H-bond interactions under initial mechanical stress. However, these stabilize or weaken over time as the complex undergoes conformational changes. Residues involved in π-cation interactions, such as Arg198 (Sirt1) and Arg53 (CXCR3), show stabilizing energy trends during MD but destabilize in SMD, reflecting their sensitivity to external forces. Phe414 (Sirt1) and Tyr205 (CXCR3), involved in π-π stacking, also exhibit destabilization, indicating that these interactions are disrupted under stretching conditions. Residues such as Leu418 (Sirt1) and Trp109 (CXCR3), which are primarily involved in hydrophobic interactions, tend to maintain their stabilizing roles in MD but show moderate destabilization during SMD. This suggests that while hydrophobic contacts contribute to the overall structural integrity, they are less resistant to mechanical stress compared to H-bonds.

The per-residue energy decomposition analysis for both Sirt1-7D and CXCR3-7D complexes reveals that hydrogen bonds and π-cation interactions are highly dynamic and responsive to mechanical stress, as evidenced by their fluctuating energy contributions during SMD simulations. In contrast, hydrophobic interactions and good contacts provide a more stable but moderate contribution to the complexes’ structural integrity. Residues such as Leu418 in Sirt1 and Trp109 in CXCR3 highlight the importance of hydrophobic contacts in maintaining stability under both MD and SMD conditions.

##### Water network analysis around 7D indicate common hydration site

**Figure 9:**
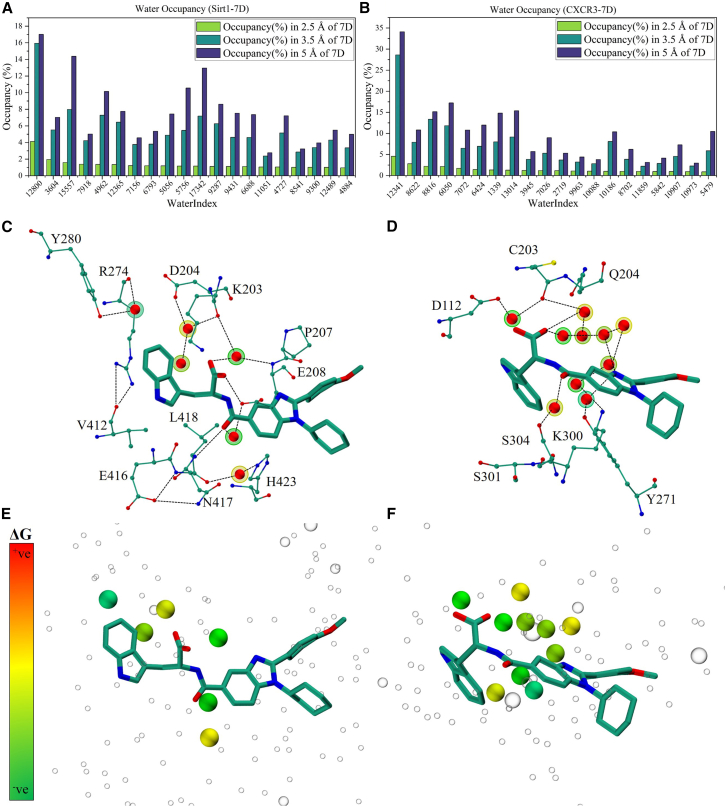
Comprehensive analysis of water-mediated interactions between 7D and its dual targets, Sirt1 and CXCR3 (A) Water occupancy profile around 7D in the Sirt1 binding pocket calculated at three distance thresholds (2.5 Å, 3.5 Å, and 5.0 Å). (B) Water occupancy profile around 7D in the CXCR3 binding pocket under the same conditions. (C) Water-mediated hydrogen bonding network between 7D and Sirt1, highlighting stable water molecules involved in indirect ligand-receptor interactions. (D) Water-mediated hydrogen bonding network between 7D and CXCR3. (E) WaterMap energetics show the distribution of favorable (green) and unfavorable (red) water molecules around 7D in the Sirt1 complex. (F) WaterMap energetics around 7D in the CXCR3 complex, demonstrating the majority of stable “happy” waters that support ligand binding. See also Figures S7 and S8.

To further elucidate the binding mechanism of 7D with Sirt1 and CXCR3, we performed an *in-depth* water network analysis using WaterMap. Water molecules play a crucial role in stabilizing ligand-receptor complexes through water-mediated interactions, and thus their contributions and occupancy around 7D were systematically evaluated (Figure 9). The distance criteria, i.e., 2.5 Å, 3.5 Å, and 5.0 Å, corresponding to the first, second, and third solvation shells, respectively, were chosen for water analysis (Figures 9A and 9B). The occupancy analysis revealed that several water molecules remained highly stable throughout the MD simulations, indicating their critical role in mediating interactions between 7D and respective binding sites. Furthermore, water-mediated interaction ,as how these highly stable water molecules are spatially distributed around 7D, contributes to maintaining its binding via water-mediated hydrogen bonds (Figures 9C and 9D). Notably, the core region of 7D, particularly involving the –COOH group, shows strong water-mediated interactions, anchoring the ligand within the binding site of both proteins. Furthermore, the -indole moiety located in the tail region of 7D also retains several stable water molecules that contribute to additional hydrogen-bonding interactions, further strengthening the ligand-receptor complex.

Subsequently, to explore the energetic profile of the solvent environment, we performed WaterMap-based free energy calculations using Schrödinger’s WaterMap tool (Figures 9E and 9F). These analyses differentiate between energetically favorable (“happy”) and unfavorable (“unhappy”) water molecules. The happy waters are generally tightly bound and contribute favorably to ligand stabilization, whereas unhappy waters are energetically unstable and can be targeted for ligand optimization by functional group addition. The WaterMap data demonstrated that the majority of the water molecules surrounding 7D are energetically favorable, especially those involved in mediating critical ligand-receptor contacts. This analysis is also useful to remove unhappy water for improved binding affinity of candidate compounds,^35^ indicating the importance of water studies.

The water occupancy analysis shows that CXCR3 exhibits slightly higher stable water occupancy around 7D compared to Sirt1, suggesting a more dynamic water network in CXCR3. These findings underscore the importance of water-mediated networks in the dual-target binding of 7D, offering valuable insights for future lead optimization strategies where water replacement or exploitation can be considered. The WaterMap analyses not only confirm the stability of 7D binding through water-mediated interactions but also highlight important opportunities for structure-based drug design.^36^ The presence of energetically favorable (“happy”) water molecules around the core and tail regions of 7D indicates that these solvent molecules play a significant role in stabilizing the complex via bridging interactions with key receptor residues. Importantly, regions where unfavorable (“unhappy”) water molecules were identified suggest that these pockets can be strategically targeted during lead optimization. By introducing polar or hydrogen-bonding functional groups into 7D that displace energetically unfavorable waters, it may be possible to enhance binding affinity toward both Srit1 and CXCR3 simultaneously. Thus, exploiting the solvent energetics and strategically modifying 7D to interact with these specific receptor environments could yield improved dual-target inhibitors with higher potency and better pharmacological profiles. Beyond supporting thermodynamic favorability, the identification of high-energy (“unhappy”) water molecules provides valuable guidance for future ligand optimization. In the Sirt1 catalytic cleft, displacement of energetically unstable hydration sites suggests opportunities to introduce polar or hydrogen bond-forming substituents capable of directly replacing these waters through stronger protein-ligand interactions. Similarly, within the CXCR3 orthosteric pocket, targeting unstable hydration clusters may allow rational extension of hydrophobic or aromatic moieties to maximize entropy gain upon water displacement. Alternatively, strategically designed functional groups could stabilize favorable water-mediated bridges that are enthalpically advantageous. Thus, hydration-site mapping not only validates current binding energetics but also offers a blueprint for structure-guided optimization to enhance affinity and selectivity. This mechanistic insight establishes a strong framework for rational optimization of 7D or its analogs as polypharmacological agents.

##### Enhanced stability of 7D in the binding sites due to consistent and high occupancy water mediated interactions

The water occupancy analysis around key atoms of the 7D ligand in the Sirt1-7D (Figure S7) and CXCR3-7D (Figure S8) complexes reveals important insights into how water molecules interact with the ligand in the binding pockets of both proteins. The comparative analysis of the water occupancy around key atoms of 7D in the Sirt1-7D and CXCR3-7D complexes reveals distinct similarities and differences in the water-mediated interactions at the atomic level, suggesting specific behavior patterns that contribute to the stability of the ligand-receptor complex in each case. For both Sirt1 and CXCR3, the oxygen atoms (O1, O2, O3) of 7D display a general trend in water occupancy, with higher water interaction observed as the distance from the atom increases (moving from the 2.5 Å 1^st^ shell to the 5.0 Å shell). However, there are noticeable differences in the extent of these interactions between the two proteins. Therefore, atom-wise characterization was performed.

O1 atom (of -COOH group) shows relatively higher water occupancy in both the complexes, particularly at larger distance thresholds (3.5 Å and 5.0 Å), indicating stable water-mediated interactions that help anchor the ligand within both the proteins. The trend in occupancy (increasing with distance) is consistent across both complexes. The O2 atom also exhibits similar behavior in both complexes, with a steady increase in water occupancy as the distance increases, although the occupancy at each shell is notably higher for CXCR3 compared to Sirt1. This suggests that O2 may have a more dynamic role in CXCR3, with more water molecules involved in stabilizing the ligand-receptor interaction at greater distances. The O3 atom, in contrast, shows much lower water occupancy, with the values being relatively consistent across both Sirt1 and CXCR3, indicating less significant water-mediated stabilization at this atom. This suggests that O3 may play a secondary role in the binding interactions of 7D with either protein, with minimal water engagement. The hydrogen atom H11, part of the -COOH group of the 7D core region, shows a pattern of water occupancy that is quite similar in both complexes, with a moderate increase in occupancy as the distance from the atom increases. This suggests that H11 plays a consistent role in interacting with water molecules, helping to stabilize the ligand-receptor complex in both Sirt1 and CXCR3. The overall behavior of H11 remains similar across the two complexes, highlighting its role in maintaining the integrity of the 7D binding.

The nitrogen atom -N4, located in the core region of 7D, shows a much lower water occupancy in both Sirt1 and CXCR3, suggesting minimal water involvement in the stabilization of this particular atom. This atom’s behavior is fairly consistent across the two proteins, indicating that the -N4 group is not a major player in water-mediated stabilization in both cases. The -indole ring of the tail region displays a significant difference between the two complexes. While in Sirt1, it shows relatively moderate water occupancy at all distance thresholds, CXCR3 exhibits a much more extensive water network surrounding the ring, particularly at the larger distance shells (3.5 Å and 5.0 Å). This suggests that the -indole ring plays a prominent role in CXCR3, with water molecules stabilizing its position more significantly compared to Sirt1. This dynamic interaction of the ring with water molecules in CXCR3 could contribute to a more stable ligand-receptor complex, with water molecules playing a crucial role in maintaining the binding integrity.

##### A common inhibition mechanism of 7D’s via substrate mimicry

**Figure 10:**
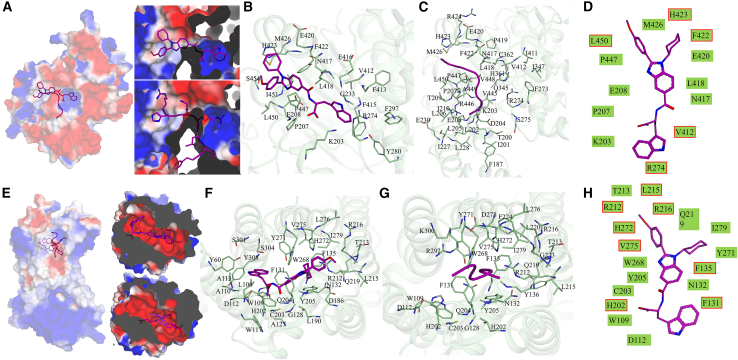
Residue occupancy profiles and substrate-mimicking interactions of 7D with Sirt1 and CXCR3 Panel (A) Docked surface views demonstrating 7D binding at the P53 substrate site of Sirt1. (B and C) Residues within 5.0 Å of 7D and P53, respectively, in Sirt1. (D) Identification of common residues contacting both 7D and P53. (E) Docked surface views show 7D binding at the PF4 substrate site of CXCR3. (F and G) Residues within 5.0 Å of 7D and PF4, respectively, in CXCR3. (H) Identification of common residues contacting both 7D and PF4. Panel F and L is also highlighting the common residues that are present in both Sirt1 and CXCR3 receptor involved in 7D binding.

To comprehensively elucidate the mechanistic basis underlying the dual-targeting ability of 7D, we have performed a detailed comparative interaction analysis, as in both cases 7D occupied the substrate binding sites. Using a combination of residue occupancy profiling and common residue mapping, we demonstrate that 7D acts by mimicking the key interactions typically made by the respective natural substrates, i.e., P53 for Sirt1 and PF4 for CXCR3, thereby supporting its role as a potential polypharmacological agent. We calculated the positional residue occupancy (residues within 5.0 Å of 7D) from the molecular dynamics simulation trajectories to understand how 7D interacts at the substrate binding sites of each receptor, whichrepresents the molecular mechanism by which 7D occludes the substrate binding site (Figure 10).

*7D Mimics Ac-P53 Interaction Network in Sirt1*: Residue-wise occupancy reveals that 7D engages a highly overlapping set of residues with P53 during molecular dynamics (Figures 10A and 10B). Notably, key residues such as Glu208 and Asn417 exhibit high occupancy values (>50%) in both 7D and P53 profiles, suggesting that 7D stabilizes itself within the substrate-binding groove in a P53-like manner. The docked surface view of 7D further confirms that 7D is positioned deeply into the binding pocket, making extensive contacts with crucial residues responsible for P53 accommodation (Figure 10C). Visualization of residues within 5.0 Å around both 7D and P53 demonstrates that the spatial distribution of interacting residues is highly similar between the two complexes and the common residues (Figures 10D–10F). This structural overlap strongly supports the idea that 7D uses the substrate-recognition features of the Sirt1 pocket, offering enhanced binding stability and potentially inhibiting enzymatic activity in a competitive manner with substrate, behaving as a substrate mimic, ensuring strong and sustained binding by occupying critical anchoring points that would normally stabilize P53.

*7D Recapitulates PF4 Binding Patterns in CXCR3*: A similar substrate-mimicking trend is observed when analyzing the binding of 7D at the CXCR3 site. Residue occupancy reveals a high degree of overlap between residues contacted by 7D and those engaged by the native substrate PF4 (Figures 10G and 10H). Importantly, residues such as Asn132, Phe135, His202, Cys203, Gln204, Tyr205, Arg212, Arg216, Tyr271, His272, Val275, and Ile279 exhibit significant occupancy levels for both 7D and PF4, indicating that 7D faithfully interacts with the key anchoring residues that maintain PF4 binding. The docked surface pose shows 7D placed within the substrate binding groove, adopting a similar spatial orientation as PF4, further visualizing the surrounding residues within 5.0 Å, demonstrating that 7D and PF4 are enveloped by highly similar residue environments during the course of binding, along with common residues (Figures 10I–10K). This finding of substrate mimicking pattern seems serendipitous in the case of 7D, however, most of the reported inhibitors generally compete with the substrate.

Furthermore, to extract the key feature of the 7D interaction pattern between both the targets, a comparative analysis was carried out. Since the common residue type was observed in both cases in direct interactions with 7D (Figures 10F and 10L). These residue types, belonging to Arg, Val, Phe, His, and Leu, were observed within 5.0 Å from 7D during molecular dynamics simulations for both proteins. In Sirt1, 7D interacts closely with residues Arg274, Val412, Phe422, His423, and Leu450, stabilizing its binding within the substrate-binding pocket. Remarkably, a similar pattern of interaction is observed in CXCR3, where 7D engages residues such as Arg212, Arg216, His202, His272, Phe131, Phe135, Val275, and Leu215, again anchoring itself within the key functional region of CXCR3. The recurrence of these specific residues in the binding interactions of 7D with both targets strongly supports the possibility of a substrate-mimicking mechanism. This shared residue environment suggests that 7D recognizes and binds similar physicochemical features across structurally different proteins, thus facilitating its dual-target engagement. The high occupancy of these residues during the simulation trajectory further emphasizes their critical role in stabilizing 7D binding. Overall, the presence of common interacting residues, viz. Arg, Val, Phe, His, and Leu across Sirt1 and CXCR3 reveal a common mechanistic insight into how 7D achieves its polypharmacological activity by exploiting conserved microenvironments within different substrate-binding pockets.

To further improve the integration of computational findings with previously reported experimental observations, a consolidated SAR summary table is added (Table S8) to summarize representative active, weakly active, and inactive analogs together with their reported Sirt1 biochemical activity profiles and corresponding computational analyses. Importantly, the observed SAR trends suggest that conserved aromatic interactions, hydrophobic stabilization, and favorable interaction geometries may contribute to the experimentally observed dual-target behavior of 7D.^2^^,^^6^ In contrast, analogs exhibiting weaker interaction networks or altered substitution patterns demonstrated comparatively reduced biological activity as previously reported. The additional CXCR3 computational analyses of representative derivatives further support the proposed dual-target interaction framework and provide structural interpretation for the observed SAR trends.

Collectively, the present computational analyses suggest that 7D exhibits polypharmacological behavior through substrate-mimetic interaction patterns across both Sirt1 and CXCR3. Rather than interacting through nonspecific surface contacts, 7D demonstrated stable engagement within the canonical substrate-associated binding regions of both targets and interacted with several functionally relevant residues identified in docking, molecular dynamics, and pharmacophore analyses. The observed interaction patterns and occupancy features provide a possible mechanistic rationale for the experimentally observed dual-target behavior of 7D and may support future optimization strategies aimed at improving dual-target interaction selectivity and stability.

## Discussion

The current study addresses a critical gap essential in modern drug discovery. The need for polypharmacological agents as antiviral that target host pathways involved in viral infections, immune dysregulation, and inflammatory responses seems a beneficial strategy to combat. Traditional single-target drugs often fail to address the complex interplay of multiple biological pathways in diseases such as viral infections, cancer, and autoimmune disorders. Hence, developing a molecule capable of modulating more than one relevant target is a pressing scientific and therapeutic need; however, due to a lack of structural features and other functional determinants, designing a polypharmacological agents is considered a *tough nut to crack*. We identified a benzimidazole derivative 7D, which is able to inhibit dual targets. We are exploring 7D ability as how it modulates via dual-target (Sirt1 and CXCR3), two distinct yet interconnected proteins in terms of function, influencing immune modulation and metabolic regulation. Our key findings demonstrated multifaceted computational arsenals, including binding free energy calculations (MM-GBSA and MM-PBSA), interaction fingerprint analysis, MD and SMD simulations, which show that 7D binds both Sirt1 and CXCR3. The binding free energy analyses indicate distinct interaction energetics levels of 7D toward CXCR3 and Sirt1. The pharmacophore and residue-wise interactions further support 7D’s binding ability on both targets rather than random cross-reactivity. The conserved pharmacophoric features such as carboxylic acid (D6), core region phenyl (R13), and phenyl (R15) from the head region of 7D, that firmly engage the substrate binding sites of both Sirt1 and CXCR3. Common interacting residue types such as Arg, Phe, Leu, and Trp were consistently observed within 5.0 Å from the 7D stable pose in both Sirt1 and CXCR3 during MD simulations (higher occupancy), suggesting that 7D binds within a conserved/common physicochemical environment at the substrate-binding site of both targets.

Interestingly, SMD results show a prolonged retention of 7D in CXCR3 during dissociation, suggesting better potential, while Sirt1 shows earlier release, a distinction that could be exploited pharmacologically. The FELs and unbinding pathways clearly differentiate the binding strengths and change in dynamics between the two targets. The per-residue decomposition energy from MD and SMD simulations revealed that, likewise, residues Arg274, Phe422, Leu418 (Sirt1), and Arg212, Phe135, Trp109 (CXCR3) provide significant stabilization to 7D in their respective binding sites. Notably, hydrophobic contacts (Leu/Trp) remained stable under stress, while charged and aromatic residues showed initial stability but destabilized under mechanical force, underscoring their dynamic role in 7D’s dual-target binding. Furthermore, the water-map analysis has provided additional valuable insights in terms of hydration dynamics around the binding sites. The water molecules identified in the binding pocket of CXCR3 appear to play a crucial role in stabilizing the ligand-protein interaction, further supporting the robust binding affinity. Water occupancy analysis around 7D reveals that the -COOH group, particularly atoms -O1, -O2, and -H11, engages in consistent and stable water-mediated interactions in both Sirt1 and CXCR3, anchoring the ligand in the binding pockets. While -N4 shows minimal water involvement, the -indole ring displays enhanced solvation in CXCR3, highlighting shared and distinct solvent-driven stabilization patterns across the two targets. These findings underscore the importance of considering water-mediated interactions in the design of polypharmacological agents, as they contribute to both the stability and dynamics of the ligand-target interactions. Notably, stable interactions of 7D perturb substrate recruitment in both cases by mimicking their endogenous substrates, i.e., P53 (in Sirt1) and PF4 (in CXCR3), thereby interfering with protein function and therefore, offering a deeper understanding of its dual-target potential.

By simultaneously targeting Sirt1, known for its role in immune suppression and viral persistence, and CXCR3, a mediator of immune cell recruitment and inflammation, 7D offers a balanced approach: enhancing antiviral immunity while preventing immune over activation.^2^ Such dual action is highly relevant for managing viral infections prone to cytokine storms, such as dengue and COVID-19. Additionally, the dual inhibitory action broadens 7D’s therapeutic scope to include cancer and autoimmune diseases as well, where Sirt1 and CXCR3 pathways are dysregulated.^2^^,^^5^^,^^6^ The present study primarily provides computational and mechanistic interpretations of the experimentally observed dual-target behavior of 7D toward Sirt1 and CXCR3. While docking, pharmacophore, molecular dynamics, steered molecular dynamics, hydration mapping, and thermodynamic energetic analyses consistently support the proposed structural hypotheses, however, due to a lack of biochemical or cellular experimental validation beyond previously reported studies, we predicted robust mechanistic insights of dual-target modulation of Sirt1 and CXCR3. By bridging metabolic regulation and immune modulation, 7D exemplifies the potential of *host-directed* therapeutics in tackling complex diseases. Our findings not only advance 7D as a lead to explore further but also pave the way to design a new class of dual-target agents based on identified determinants. This study provides an experimental-basis computational framework for understanding dual-target interaction behavior that support future rational design strategies for the discovery of polypharmacological small molecules on the same or diverse biological pathways.

### Limitations of the study

The proposed hypotheses are supported by our previously reported phenotypic, activity-based, and *in vivo* experimental observations. However, based on previous findings, the hypothesized current computational outcomes required direct experimental validation of 7D interactions and critical determinants of its polypharmacological behavior that may guide future experimental validation for dual-target drug discovery efforts.

## Resource availability

### Lead contact

Further information and requests for resources should be directed to and will be fulfilled by the lead contact, Shailendra Asthana (sasthana@thsti.res.in).

### Materials availability

This study did not generate new unique reagents.

### Data and code availability

• Data: Crystal structures used in this study were obtained from the Protein Databank (PDB). Docked poses, molecular dynamics (MD), and steered molecular dynamics (SMD) simulations input files generated during this study will be made available by the lead contact upon reasonable request.
• Code: Computational analyses were performed using a combination of commercial and open-source software. Structure preparation, MCS docking, ePharmacophore modeling, and BPMD analyses were carried out using licensed versions of the Schrödinger software suite. MD and MM-GBSA/MM-PBSA free-energy calculations were performed using AMBER22. All visualization and trajectory analyses were conducted using VMD and PyMOL. Graphs and figures were prepared using OriginPro 2025b (Learning Edition). No custom code was developed for this study.
• Additional Information: Any additional information required to reproduce or re-analyze the results reported in this study is available from the lead contact upon reasonable request.

## Acknowledgments

The authors would like to acknowledge 10.13039/501100001407DBT and 10.13039/100026280BRIC-10.13039/501100020622THSTI for providing support, research opportunities and computational facilities. We also acknowledge Translational Research Program (10.13039/100029424TRP) of 10.13039/501100020622THSTI for funding and support and NNP-10.13039/501100001407DBT (grant no. BT/PR40189/BTIS/137/50/2022). Authors are thankful to the ICMR for the funding (ICMR/14/1923/SGP-2023 25–26), and ICMR P521 (ICMRCAREP-2023-0000176).

## Author contributions

S.A. proposed and designed the study. K.B.L. performed all the experiments and computational analyses. K.B.L., D.R.M., and S.A. were involved in the discussion and analysis. K.B.L. and S.A. wrote and revised the manuscript. All authors have read and approved the manuscript.

## Declaration of interests

The authors declare no competing financial interests.

## STAR★Methods

### Key resources table

**Table:** 

REAGENT or RESOURCE	SOURCE	IDENTIFIER
**Software and algorithms**		

Desmond	Schrödinger, Inc.	https://www.schrodinger.com/platform/products/desmond/
Protein Preparation Wizard	Schrödinger, LLC, New York, NY, 2020.	https://www.schrodinger.com/
OPLS4 force field	Lu et al.^37^	https://doi.org/10.1021/acs.jctc.1c00302
Phase	Schrödinger, Inc.	https://www.schrodinger.com/platform/products/phase/
Amber22	Case et al.^38^	https://www.ambermd.org/
ff14SB force field	Maier et al.^39^	https://doi.org/10.1021/acs.jctc.5b00255
Steered Molecular Dynamics simulation	Park et al.^40^	https://doi.org/10.1063/1.1590311
Weighted Histogram Analysis Method	Kumar et al.^41^	https://doi.org/10.1002/jcc.540161104

### Method details

#### Binding pose metadynamics

To identify the correct binding poses of 7D within Sirt1 and CXCR3, we conducted Binding Pose Metadynamics (BPMD) simulations using Schrödinger’s Desmond module.^42^ For CXCR3*,* the previously obtained docked pose of 7D was utilized.^2^ However, for Sirt1, the N-terminal region was added to provide a more complete representation of the binding cavity. This addition allowed us to achieve a precise docking pose of 7D within Sirt1 using the Maximum Common Substructure (MCS) docking method^43^ available in Schrödinger suite. Additionally, Figure S9 illustrates the binding pose comparison and clustering analysis of the 7D molecule against the CXCR3 receptor structure (PDB ID: 8K2W), available in the Protein Data Bank. In the left panel, blind docking of 7D reveals two major clusters: Cluster 1 (green) and Cluster 2 (pink). The 7D docked pose (green sticks) overlaps closely with the co-crystallized antagonist AMG487 (blue sticks) in Cluster 1, highlighting a strong alignment between the two binding modes. In contrast, Cluster 2 aligns with the binding site of another known CXCR3 antagonist, SCH546738 (pink surface and sticks). The right panel shows the docking score distribution for all docked 7D poses. Most poses (green dots) belong to Cluster 1, exhibiting better binding affinities, while Cluster 2 contains fewer poses (pink dots) with comparatively weaker affinities. Histograms and probability density curves beside the scatter plot summarize the docking score distribution for each cluster.^44^ This analysis validates that the 7D molecule predominantly occupies the AMG487 binding site on CXCR3, supporting its structural relevance and suggesting a similar binding mechanism.

Once the docking poses were prepared, BPMD simulations were set up to evaluate the stability of these poses under dynamic conditions. The protein-ligand complexes were first prepared using Schrödinger’s Protein Preparation Wizard,^45^ and each system was solvated in a cubic box with a 12.0 Å buffer filled with TIP3P water molecules. Counterions were added to neutralize the system, and the OPLS4 force field^37^ was applied to both the protein and ligand to ensure accurate representation of molecular interactions. The metadynamics simulations were performed for 10 ns on each complex, with 10 independent trial runs per complex to ensure reproducibility and statistical robustness. During BPMD, the ligand's root-mean-square deviation (RMSD) relative to its initial docked position was used as the collective variable (CV) to monitor pose stability. Before initiating the BPMD simulations, each system underwent energy minimization to relax the structure, eliminate steric clashes, and gradually equilibrate to 300 K. The results of the BPMD simulations were analyzed using two key metrics: the Persistence Score (PersScore) and the Pose Score (PoseScore). The PersScore reflects the duration the ligand remains in its initial binding pose during the simulation, with higher values indicating greater stability. The PoseScore quantifies the overall stability of the ligand’s binding mode by measuring deviations from the initial pose over time, with lower scores indicating more stable binding.

#### ePharmacophore hypothesis

In this study we have utilized the complex structures of Sirt1-7D and CXCR3-7D to generate the possible ePharmacophore hypothesis from the complex structures using Phase^46^ application in Schrödinger. The e-pharmacophore approach employed here quantifies these interactions by calculating the interaction energy for each feature, allowing for a detailed understanding of the ligand's binding affinity and specificity. The Phase e-pharmacophore modelling represents the interaction profiles of a 7D with two targets, Sirt1 and CXCR3.

#### Interaction fingerprint analysis methodology

The interaction fingerprint analysis of 7D within Sirt1 and CXCR3 was carried out.^47^ The Interaction Fingerprint (IFP) tool in Maestro was used to detect and categorize interactions between the ligand and surrounding protein residues, with three primary distance thresholds: 2.5 Å for direct interactions (e.g., hydrogen bonds, hydrophobic contacts), 3.5 Å for moderate interactions (e.g., van der Waals forces), and 5.0 Å for distant interactions (e.g., weak electrostatic or polar interactions). Various interaction types were considered, including hydrophobic interactions, hydrogen bonding, ionic interactions, and aromatic stacking. The interaction profiles were visualized using the Ligand-Protein Interaction Diagram in Maestro, highlighting key residues involved in important interactions and binding motifs. These profiles were compared between Sirt1 and CXCR3 to identify conserved and distinct binding patterns. Finally, residue contribution analysis identified which residues were crucial for stabilizing the ligand-receptor complex, providing a deeper understanding of the binding behaviour and the specific interactions that contribute to the affinity and specificity of ligand 7D within both targets.

#### All-atom MD simulations to assess the binding stability of 7D with Sirt1 and CXCR3

For assessing the stability of the 7D, we used the full Sirt1 structure complexed with 7D and a modelled CXCR3 structure complexed with 7D for simulations. We employed the Amber ff14SB force field^39^ with the TIP3P water model. The ligand 7D was parameterized using the General Amber Force Field (GAFF2). Atom types were assigned using the Antechamber module of AmberTools, and partial atomic charges were calculated using the AM1-BCC method. Missing bonded and dihedral parameters were generated using the parmchk2 utility, and the resulting topology and coordinate files were prepared using the tleap module. The compatibility between ff14SB and GAFF2 force fields was ensured during system assembly. The simulations were conducted using the Amber22 package,^38^ with all-atom molecular dynamics simulations. The system was prepared by placing the protein in a cubic water box with a minimum 12 Å buffer from the box edges, solvated with explicit water molecules (TIP3P) and counter-ions. Energy minimization was performed in three steps: (i) protein minimization with harmonic restraints on all non-hydrogen atoms (1 kcal/mol/Å^2^) using 2000 steepest descent and 8000 conjugate gradient steps; (ii) minimization of the full system with additional solute restraints (2 kcal/mol/Å^2^) using the same protocol; and (iii) unrestrained minimization of the entire system to allow full relaxation.^5^

After minimization, the system was heated from 0 to 300 K over 10 ps under the NVT ensemble using a 2 fs time step, with solute restraints that were released after 9000 steps. Density equilibration was then performed at 1 bar under the NPT ensemble for 25,000 steps while maintaining 300 K, with positional restraints on the solute. Production MD simulations were conducted in triplicate for 500 ns with a 2 fs time step. Temperature was regulated at 300 K using a Langevin thermostat, and pressure was maintained at 1.013 bar using the Nosé-Hoover-Langevin piston. Electrostatics were treated with PME (1.0 Å grid spacing, 12.0 Å cutoff), employing periodic boundary conditions and long-range corrections for electrostatic and Lennard-Jones interactions.^5^

The MD simulations were monitored throughout the production phase by analyzing the root mean square deviation (RMSD), root mean square fluctuation (RMSF), and interaction profiles to assess the stability and binding behaviour of the Sirt-7D and CXCR3-7D complexes.^48^^,^^49^ Also, PCA analysis was performed using CPPTRAJ to extract the principal components from the 500 ns MD simulation trajectories of the protein-ligand complexes (Sirt1-7D and CXCR3-7D). The analysis was focused on the backbone atoms (C-α) to capture the dominant conformational fluctuations.^35^ The covariance matrix was computed, and the first two principal components were visualized to explore the major motions in the systems. Further, the SHAM module of GROMACS was used to plot the Free Energy Landscape (FEL) of the protein-ligand complexes. FEL was constructed based on the RMSD and RMSF of the systems over the 500 ns production phase. Additionally, free energy decomposition was carried out using MM/GBSA and MM/PBSA methods via CPPTRAJ. The binding free energies between the ligands (7D) and proteins (Sirt1, CXCR3) were calculated, and the contributions of individual energetic components were assessed. Entropy calculations were also performed to evaluate the entropic contributions to the binding free energy.

#### Steered molecular dynamics (SMD) simulation and umbrella sampling

To explore the exit pathway and binding residues for enroute to dissociation of 7D from both Sirt1 and CXCR3, we performed Steered Molecular Dynamics simulations multiple times with varying parameters to optimize the pulling force and distance.^40^^,^^50^ These simulations were conducted using Amber22 with the ff14SB force field for proteins and the TIP3P water model. The reaction coordinate was defined by the centre of mass (COM) of either the protein or binding site residues, depending on the system being investigated. For Sirt1, we tested multiple pulling parameters considering the COM of the protein. The configurations tested were: (1) Force constant = 400 kcal/mol, Pull Distance = 50 Å, (2) Force constant = 350 kcal/mol, Pull Distance = 50 Å, (3) Force constant = 300 kcal/mol, Pull Distance = 40 Å, and (4) Force constant = 400 kcal/mol, Pull Distance = 40 Å (Figures S10A–S10D). In addition to the COM of the Sirt1 protein, we also considered the COM of Sirt1 binding site residues to assess how different regions of the protein influence the unbinding pathway. The parameters tested here included: (1) Force constant = 400 kcal/mol, Pull Distance = 50 Å, (2) Force constant = 350 kcal/mol, Pull Distance = 50 Å, (3) Force constant = 300 kcal/mol, Pull Distance = 40 Å, and (4) Force constant = 400 kcal/mol, Pull Distance = 40 Å (Figures S10E–S10H). After evaluating the results, the Force constant = 400 kcal/mol and Pull Distance = 40 Å configuration with reaction coordinates defined with COM of binding site residues proved to be most effective, ensuring stable unbinding of 7D without significant protein conformational changes.

For CXCR3, a transmembrane protein, we adopted a different approach due to the embedded helices in the membrane region. We considered the COM of the CXCR3 protein, with pulling parameters of Force constant = 400 kcal/mol and Pull Distance = 60 Å, to pull the ligand out of the transmembrane region successfully (Figure S11A). This configuration allowed us to extract 7D from the holo space between the transmembrane regions. Additionally, we tested pulling based on the COM of binding site residues with the parameters (1) Force constant = 500 kcal/mol, Pull Distance = 30 Å and (2) Force constant = 500 kcal/mol, Pull Distance = 50 Å (Figures S10B and S10C). However, these resulted in the ligand coming out from between the transmembrane helices, which is unrealistic since these helices are embedded in the membrane. Therefore, these configurations were discarded.

Following SMD, the most stable and physically plausible unbinding pathways were selected for umbrella sampling (US) simulations using AMBER22. The centre-of-mass (COM) distance of the protein or binding-site residues was used as the reaction coordinate, with harmonic biasing potentials applied across multiple windows to ensure adequate sampling. Each window was simulated for 5 ns. The distance distributions from all windows were combined using the Weighted Histogram Analysis Method (WHAM) to construct the potential of mean force (PMF), yielding a detailed energetic profile of ligand unbinding.^41^

### Quantification and statistical analysis

There are no quantification or statistical analyses to include in this study.
