# Supplementary material for: Harnessing the structural determinants amenable for polypharmacological behavior of 7D against Sirt1 and CXCR3
Source: iScience. 2026 Jun 29;29(7):116635. doi: 10.1016/j.isci.2026.116635 (PMC13378322; doi:10.1016/j.isci.2026.116635)

iScience, Volume *29*

Supplemental information

Harnessing the structural determinants amenable for polypharmacological behavior of 7D against Sirt1 and CXCR3

Kiran Bharat Lokhande, Dhani Ram Mahato, and Shailendra Asthana

Supplementary Figures:

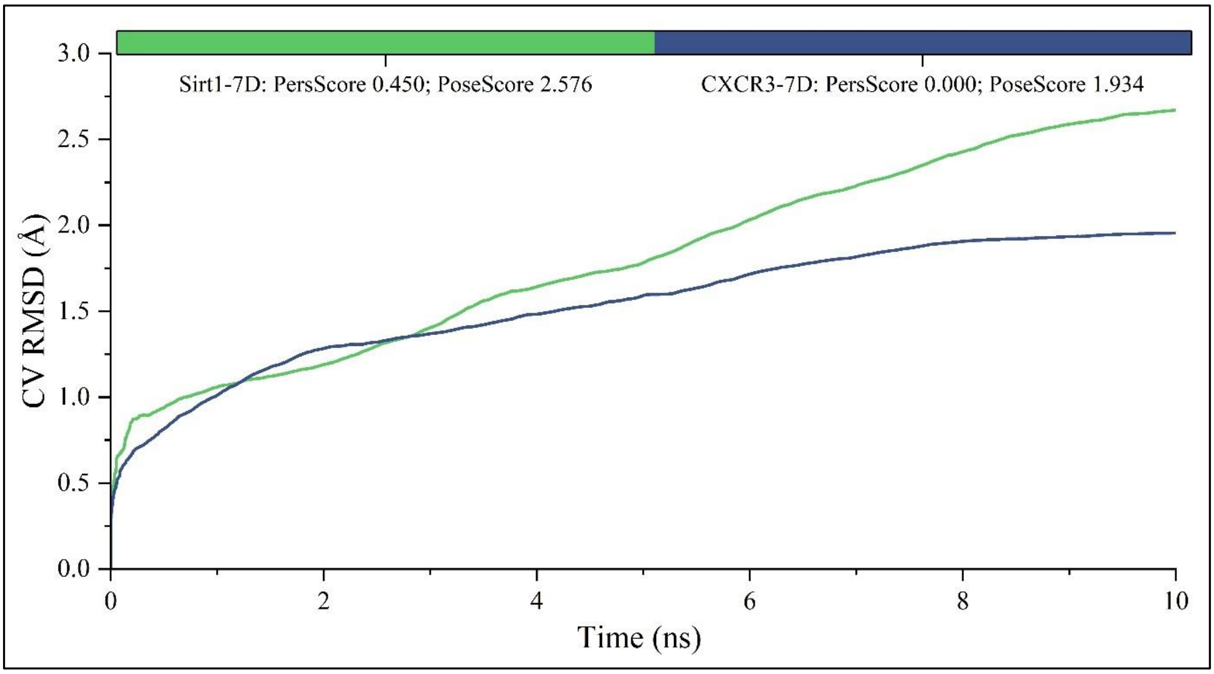

Figure S1: Binding Pose Metadynamics analysis of compound 7D with Sirt1 and CXCR3. The RMSD profiles represent the deviation of the ligand's binding pose over a 10 ns simulation. The green line corresponds to the Sirt1-7D complex, while the blue line represents the CXCR3-7D complex. Sirt1-7D exhibits a gradual increase in RMSD, reaching approximately 2.6 Å, indicating moderate binding stability with conformational shifts. In contrast, CXCR3-7D shows a slower RMSD increase, plateauing bellow 2.0 Å, suggesting a more stable binding pose.

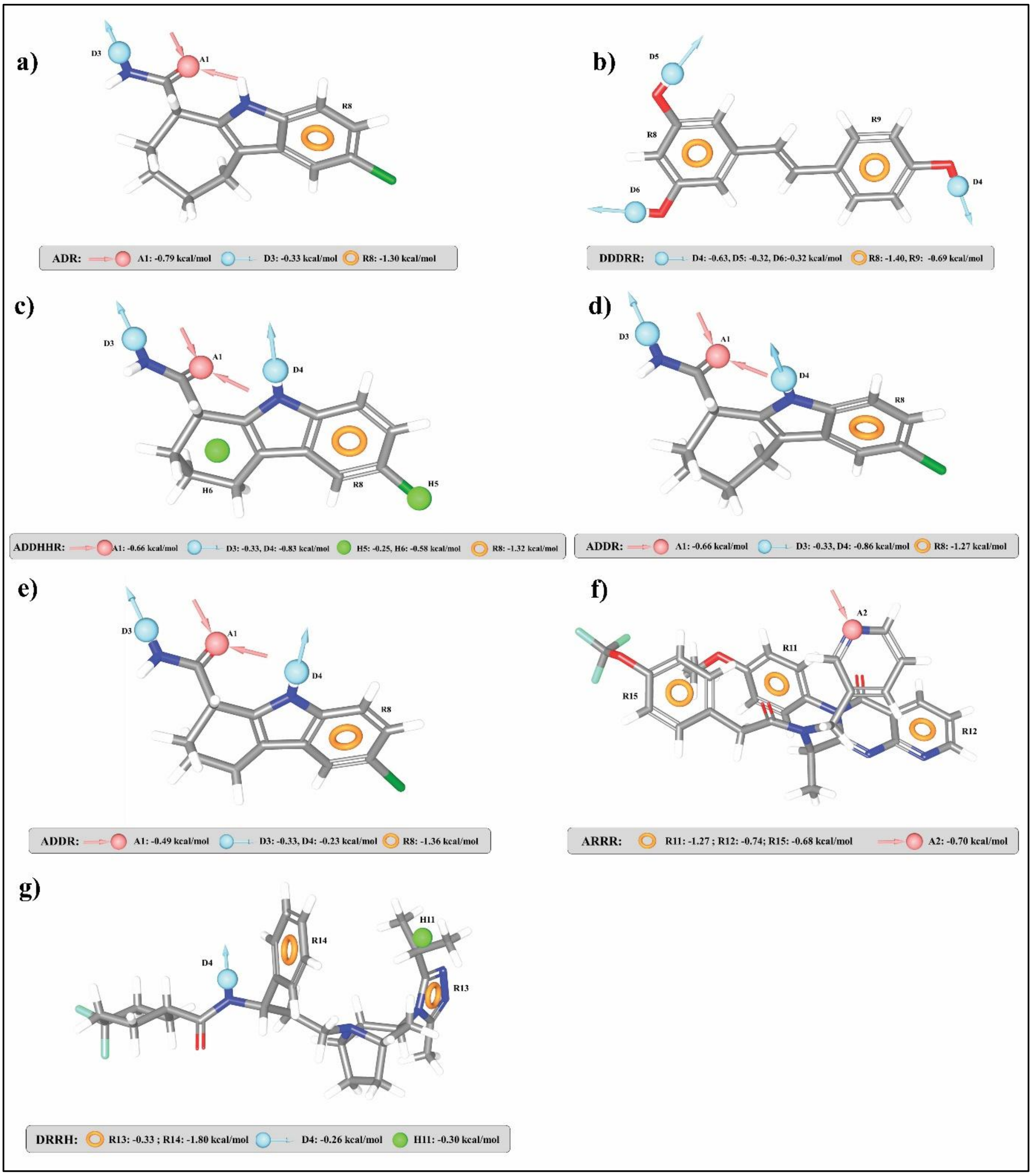

Figure S2: The e-pharmacophoric models represent the functionality arrangement and the available features on (a) Sirt1-EX527* complex, (b) Sirt1-Resveratrol complex, (c) Sirt2-EX243 complex, (d) Sirt2-EX527* complex, (e) Sirt3-EX527 complex, (f) CXCR3-AMG487 complex, and (g) CCR5-MRV complex.

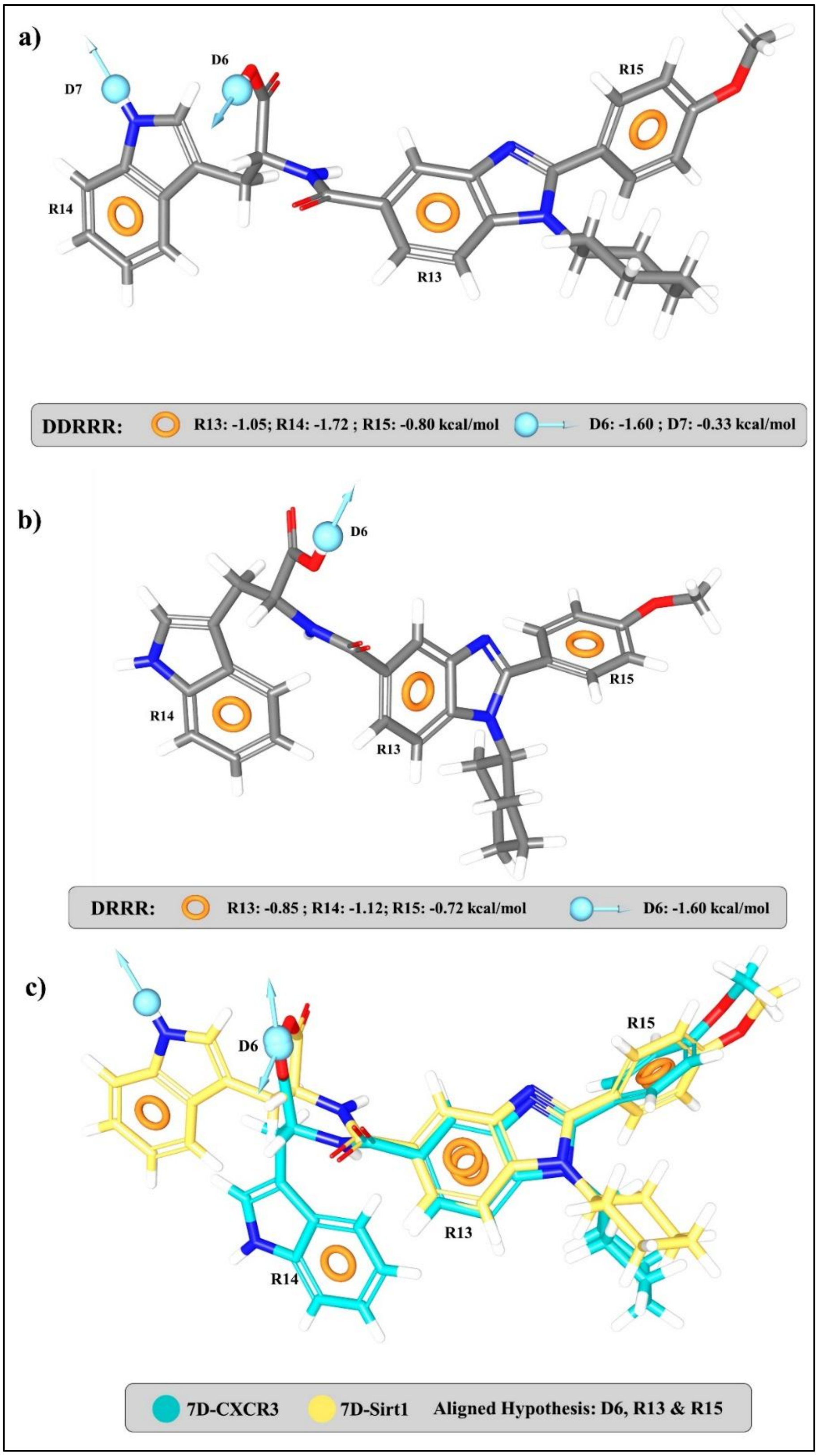

Figure S3: The e-pharmacophoric models represent the functionality arrangement and the available features on (a) Sirt1-7D complex, (b) CXCR3-7D complex, and (c) common features that are overlapping on 7D when aligned with Sirt1-7D and CXCR3-7D complex.

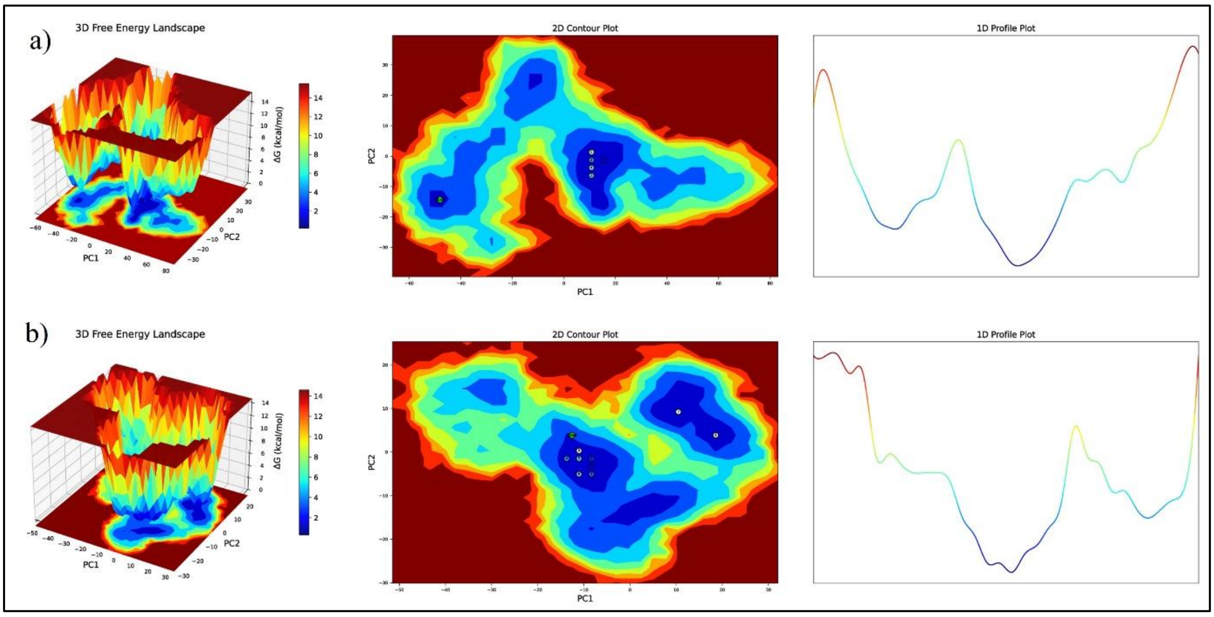

Figure S4: Free Energy Landscape (FEL): The FEL plots represent the conformational space sampled during molecular dynamics simulations of the protein-ligand complexes. The global minima states, indicating the most thermodynamically stable conformations, were identified and extracted for both Sirt1-7D and CXCR3-7D complexes. The starting minima of each complex highlighted in green circle whereas the global minima of the trajectory highlighted in deepest blue coloured circle. These global minima structures were subsequently used for detailed residue distribution and interaction fingerprint analysis.

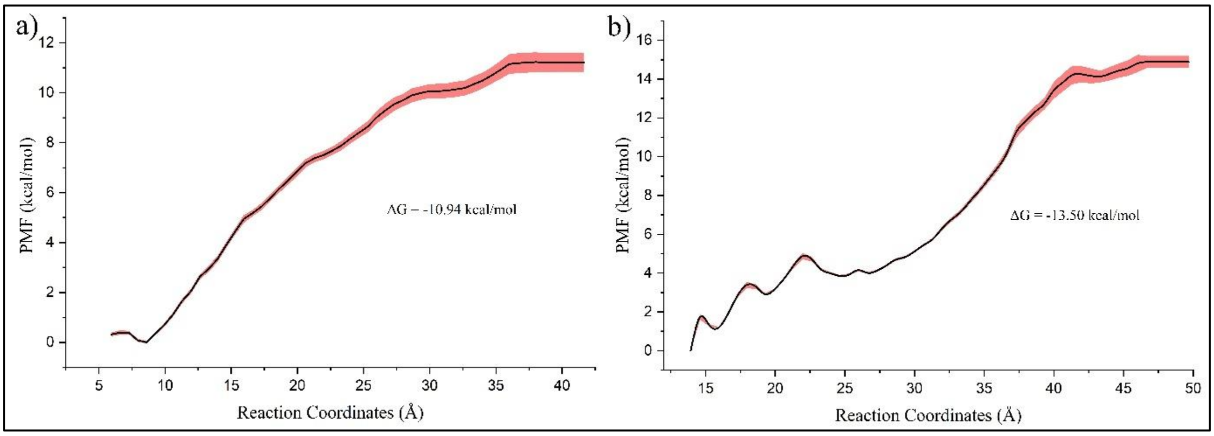

Figure S5: Potential of mean force (PMF) plot for the unbinding of 7D from (a) Sirt1, showing a final dissociation energy of -10.94 kcal/mol; and (b) from CXCR3, with a final dissociation energy of -13.50 kcal/mol.

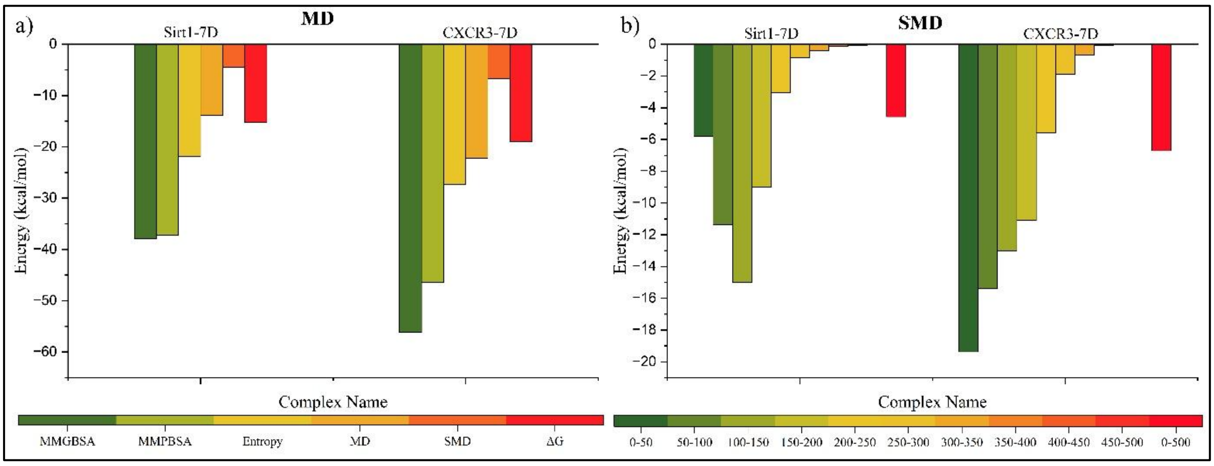

Figure S6: **Comparative binding energy analysis of 7D.** (a) The figure depicts MM-GBSA and MM-PBSA binding free energy calculations, entropy contributions, 7D contribution with Sirt1 and CXCR3 from MD simulation, and steered molecular dynamics, and free energy (ΔG) values for both complexes. (b) Steered molecular dynamics energy analysis of ligand 7D with Sirt1 and CXCR3. The figure depicts energy contribution of 7D during different time frames (0–500), illustrating the dissociation behaviour of 7D under external force.

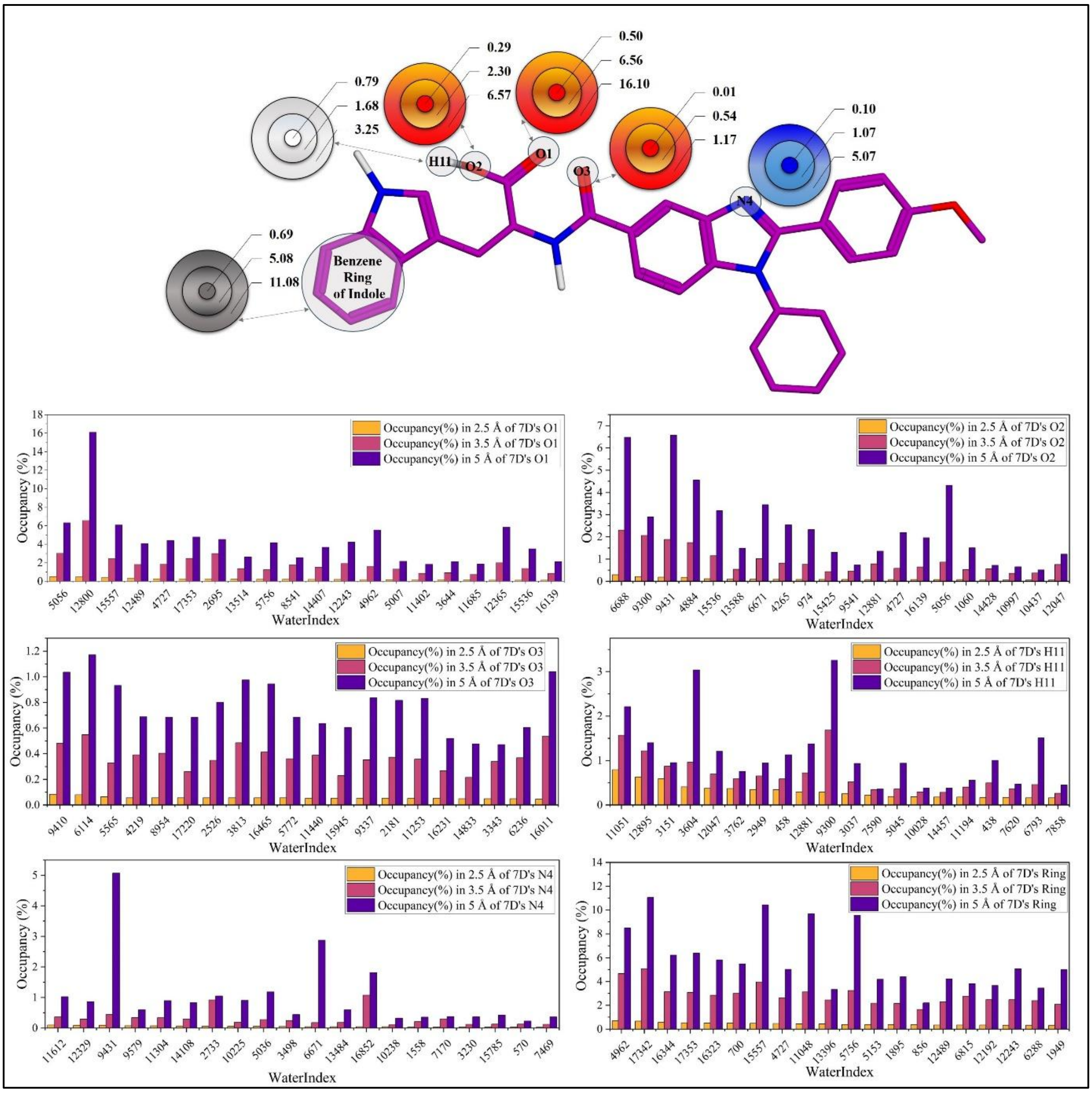

Figure S7: Water Occupancy Around Specific Atoms of 7D in the Sir1 Complex. Water occupancy profile for specific atoms of 7D in the Sirt1-7D complex at distance thresholds of 2.5 Å, 3.5 Å, and 5.0 Å for O1, O2, O3, H11, N4, and the indole ring structure.

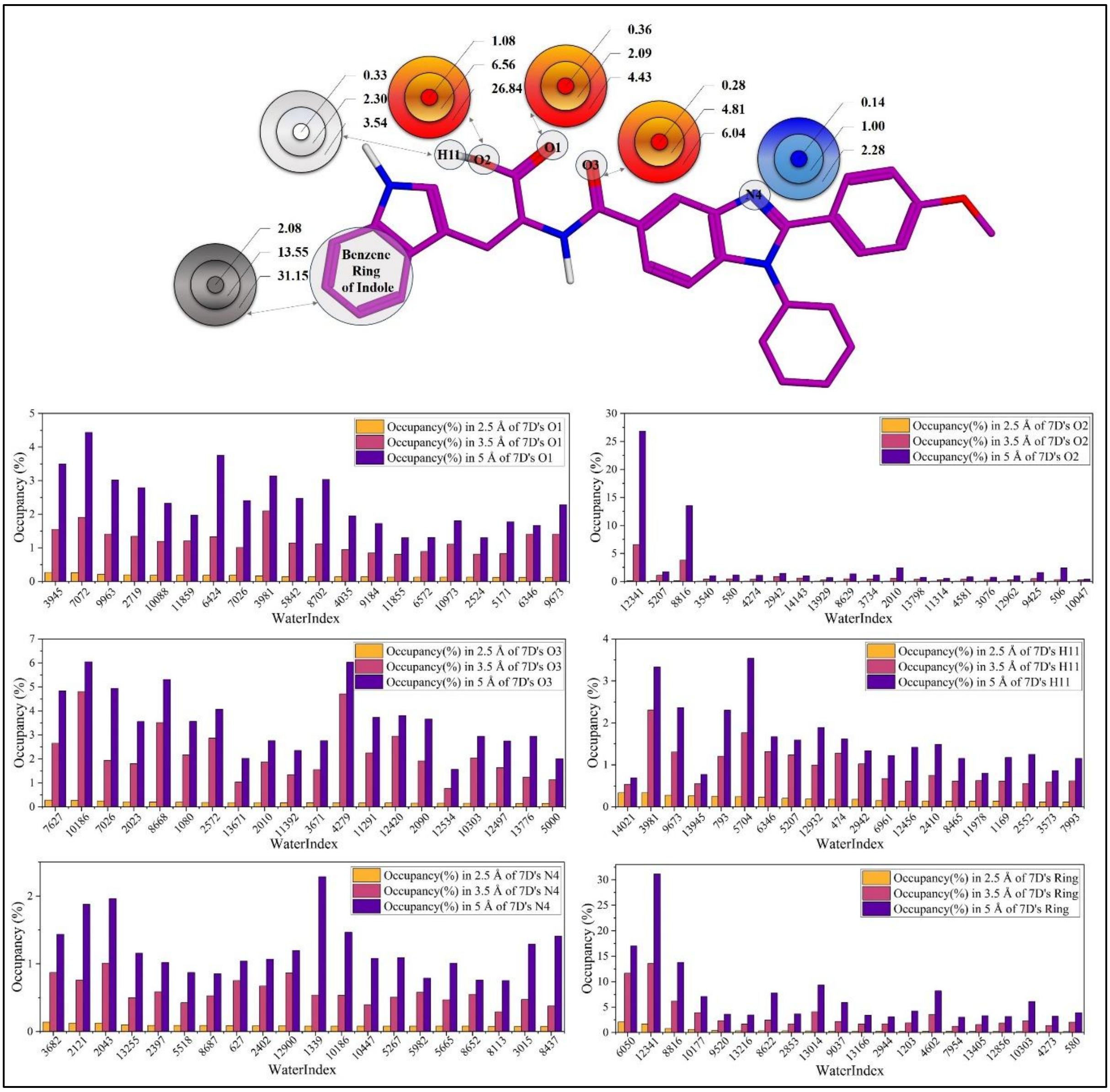

Figure S8: Water Occupancy Around Specific Atoms of 7D in the CXCR3 Complex. Water occupancy profile for specific atoms of 7D in the Sirt1-7D complex at distance thresholds of 2.5 Å, 3.5 Å, and 5.0 Å for O1, O2, O3, H11, N4, and the indole ring structure.

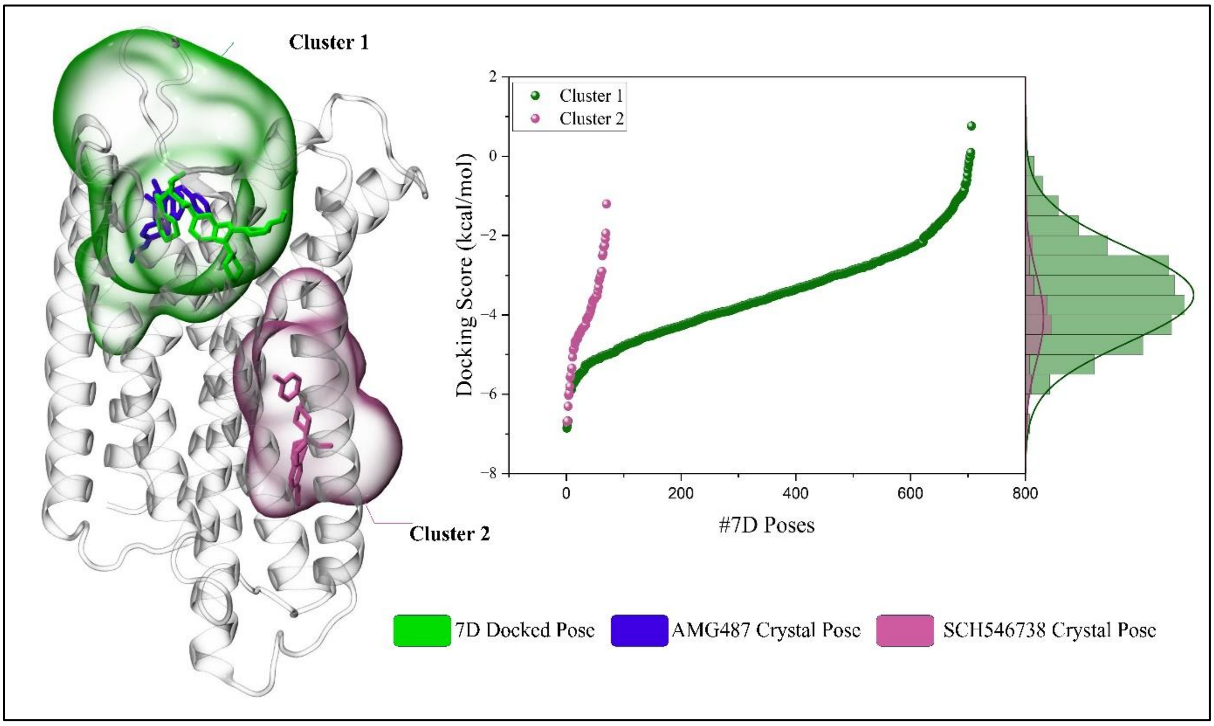

Figure S9: Blind docking analysis of 7D molecule against CXCR3 receptor structure (PDB ID: 8K2W). The left panel shows the CXCR3 receptor (grey cartoon) with clustered binding poses. Cluster 1 (green surface) represents the dominant binding region, where the docked 7D molecule (green sticks) overlaps significantly with the co-crystallized antagonist AMG487 (purple sticks). Cluster 2 (pink surface) is occupied by SCH546738 (pink sticks), another known selective CXCR3 antagonist. The right panel displays the distribution of docking scores for the 7D docked poses. Green and pink dots represent poses classified into Cluster 1 and Cluster 2, respectively. Most of the docked 7D poses fall into Cluster 1 with favorable binding scores, suggesting strong structural alignment with the AMG487 binding pocket. A marginal population is seen in Cluster 2 with slightly less favorable docking scores. Histograms and density curves summarize the score distributions across clusters.

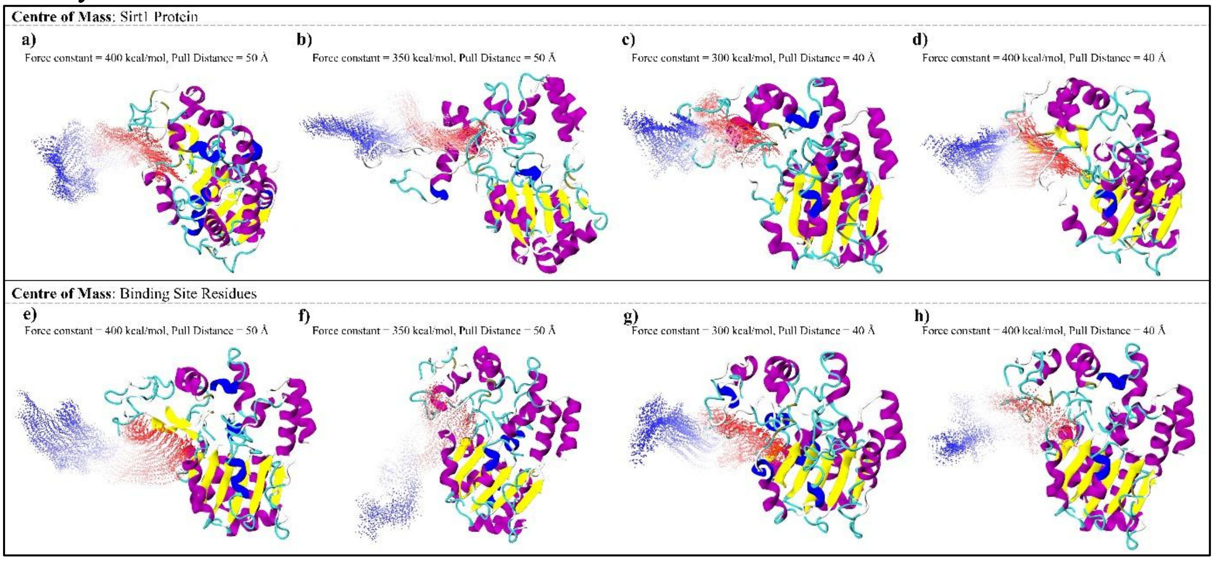

Figure S10: Steered Molecular Dynamics (SMD) simulations exploring the unbinding pathways of ligand 7D from SIRT1 under various force constants and pulling distances. **Panels (a–d):** SMD simulations of the SIRT1-7D complex with pulling applied using the centre of mass (COM) of the entire SIRT1 protein. The tested configurations include: (a) Force constant

= 400 kcal/mol, Pull Distance = 50 Å; (b) Force constant = 350 kcal/mol, Pull Distance = 50 Å; (c) Force constant = 300 kcal/mol, Pull Distance = 40 Å; (d) Force constant = 400 kcal/mol, Pull Distance = 40 Å. **Panels (e–h):** SMD simulations of SIRT1-7D with pulling applied using the COM of the binding site residues. Configurations tested: (e) Force constant = 400 kcal/mol/Å², Pull Distance = 50 Å; (f) Force constant = 350 kcal/mol, Pull Distance = 50 Å; (g) Force constant = 300 kcal/mol, Pull Distance = 40 Å; (h) Force constant = 400 kcal/mol, Pull Distance = 40 Å. Among these, panel (h) represents the most effective setting for SIRT1, ensuring stable unbinding of ligand 7D without inducing significant conformational changes in the protein.

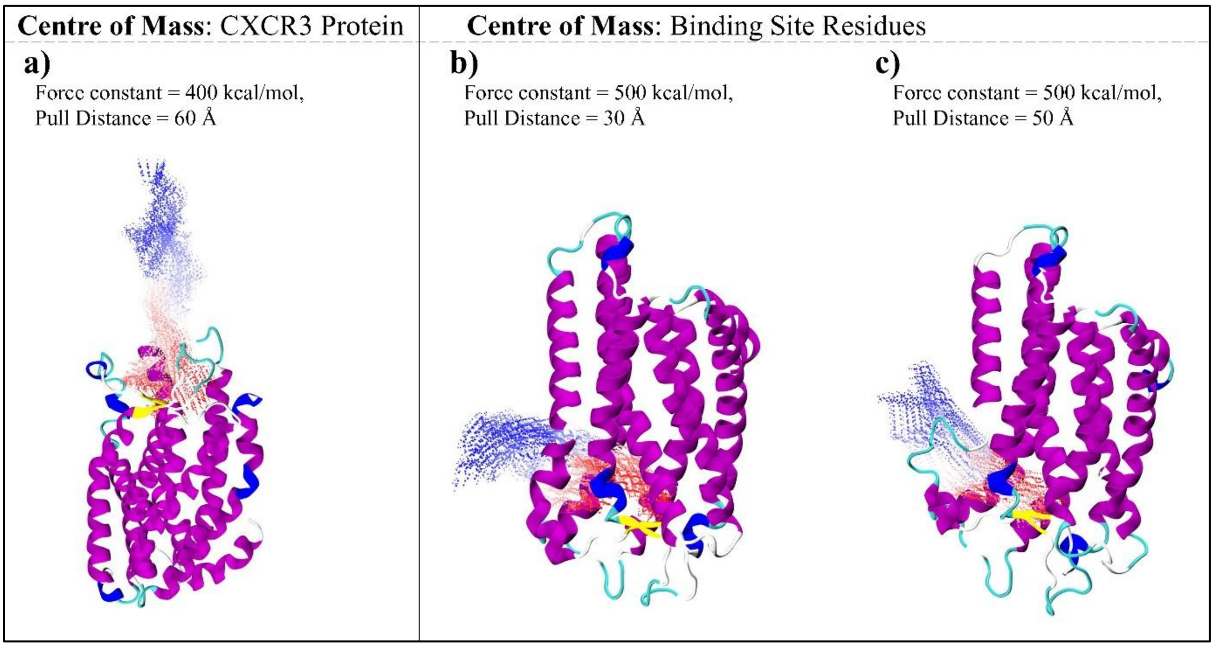

Figure S11: SMD simulations of CXCR3-7D complex, considering the transmembrane nature of CXCR3: (**a**) Pulling based on the COM of the entire CXCR3 protein with a Force constant = 400 kcal/mol/Å² and Pull Distance = 60 Å, enabling successful extraction of 7D from the transmembrane region. (**b**) Pulling based on the COM of binding site residues with Force constant = 500 kcal/mol/Å², Pull Distance = 30 Å. (**c**) Pulling based on the COM of binding site residues with Force constant = 500 kcal/mol/Å², Pull Distance = 50 Å. Pulling from binding site residues in (b) and (c) resulted in unrealistic ligand escape through transmembrane helices, whereas configuration (a) provided a more realistic unbinding pathway through the extracellular region.

Supplementary Tables

Table S1: Structural Quality Assessment and Preprocessing Details of PDB Structures Used in This Study.

PDB ID	Resolution (Å)	Coverage (A.A. Length)	R-Value (Observed)	Missing Region	Modeling/Remark
4ZZI	2.73	356	0.193	503-640	Used structure from Gly183-Cys502
4ZZJ	2.74	356	0.184	503-640	Used structure from Gly183-Cys502
5BTR	3.20	397	0.209	157-173	Modelled using Prime
8K2W	3.00	507	EM Structure	None	N/A

Supplementary Table S2: Molecular Docking and MM/GBSA Analysis of 7D and Other Known Inhibitors against Sirt1 and CXCR3.

Target Name	Compound Name	Docking Energy (kcal/mol)	MM/PB(GB)SA (kcal/mol)
Sirt 1	EX527 (control)	-7.8	-63.58
Sirt 1	7D	-8.81	-72.08
Sirt1	Tenovin-1	-6.218	-30.68
CXCR3	AMG487 (control)	-5.57	-66.37
CXCR3	7D	-8.06	-62.58
CXCR3	Melatonin	-5.365	-32.97

Table S3: Summary of residues involved in the binding site of 7D for SIRT1 and CXCR3 with interaction types and properties for the initial docked conformations of both the receptor with 7D.

Complex Name	Residues Involved in Binding Site (5Å)	Interaction Type	Bond Distance (Å)	Type of Residues
**Sirt1-7D**	Pro207	-	-	Charged (-Ve)
	Glu208	-	-	Hydrophobic
	Ala262	-	-	Hydrophobic
	Ser265	-	-	Polar
	Ile270	-	-	Hydrophobic
	Pro271	-	-	Hydrophobic
	Asp272	H-Bond	1.83	Charged (-Ve)
	Phe273	2 Pi-Pi Stacking	3.57, 3.98	Hydrophobic
	Arg274	-	-	Charged (+Ve)
	Tyr280	H-Bond	1.95	Hydrophobic
	Phe297	-	-	Hydrophobic
	Ile316	-	-	Hydrophobic
	Gln345	-	-	Polar
	Asn346	-	-	Polar

	Ile347	-	-	Hydrophobic
	Asp348	-	-	Charged (-Ve)
	His363	-	-	Polar
	Ile411	-	-	Hydrophobic
	Val412	-	-	Hydrophobic
	Phe413	-	-	Hydrophobic
	Phe414	-	-	Hydrophobic
	Gly415	-	-	Hydrophobic
	Glu416	-	-	Charged (-Ve)
	Asn417	-	-	Polar
	Leu418	-	-	Hydrophobic
	His423	-	-	Polar
	Val445	-	-	Hydrophobic
	Arg446	-	-	Charged (+Ve)
	Pro447	-	-	Hydrophobic
	Leu450	-	-	Hydrophobic
**CXCR3-7D**	Tyr60	-	-	Hydrophobic
	Leu106	-	-	Hydrophobic
	Trp109	-	-	Hydrophobic
	Ala110	-	-	Hydrophobic
	Asp112	-	-	Charged (-Ve)
	Ala113	-	-	Hydrophobic
	Trp117	-	-	Hydrophobic
	Ala127	-	-	Hydrophobic
	Gly128	-	-	Hydrophobic
	Phe131	-	-	Hydrophobic
	Asn132	-	-	Polar
	Phe135	-	-	Hydrophobic
	Asp186	-	-	Charged (-Ve)
	His202	-	-	Polar
	Cys203	-	-	Hydrophobic
	Gln204	-	-	Polar
	Tyr205	Pi-Pi Stacking	4.23	Hydrophobic
	Arg212	Pi-Cation	6.12	Charged (+Ve)
	Thr213	-	-	Polar
	Leu215	-	-	Hydrophobic
	Arg216	-	-	Charged (+Ve)
	Gln219	-	-	Polar
	Trp268	-	-	Hydrophobic
	Tyr271	-	-	Hydrophobic
	His272	-	-	Polar
	Val275	-	-	Hydrophobic
	Leu276	-	-	Hydrophobic
	Ile279	-	-	Hydrophobic
	Ser301	-	-	Polar
	Ser304	-	-	Polar
	Tyr308	-	-	Hydrophobic

Table S4: Interaction fingerprint analysis of Sirt1 with 7D and CXCR3 with 7D (docked pose) at different binding site region.

**Sirt1-7D-Docked Pose**	**Distance from 7D (2.5 Å)**	**Residue Type**	**7D Region**	**Distance from 7D (3.5 Å)**	**Residue Type**	**7D Region**	**Distance from 7D (5 Å)**	**Residue Type**	**7D Region**
	Ala262	Hydrophobic	Tail	Pro207	Hydrophobic	Head	Glu208	Charged (-Ve)	Head
	**Asp272**	Charged (-Ve)	Tail	Ile270	Hydrophobic	Tail	Ser265	Polar	Head
	**Phe273**	Hydrophobic	Tail	Pro271	Hydrophobic	Tail	Ile316	Hydrophobic	Tail
	**Tyr280**	Hydrophobic	CT	Arg274	Charged (+Ve)	CT	Ile411	Hydrophobic	Core
	Asn346	Polar	Tail	Phe297	Hydrophobic	Core	His423	Polar	Head
	Ile347	Hydrophobic	Tail	Gln345	Polar	Tail			
	Val412	Hydrophobic	Core	Asp348	Charged (-Ve)	Tail			
	Phe414	Hydrophobic	Core	His363	Polar	Core			
	Gly415	Hydrophobic	Head	Phe413	Hydrophobic	Core			
	Glu416	Charged (-Ve)	Head	Asn417	Polar	Head			
	Leu418	Hydrophobic	Head	Arg446	Charged (+Ve)	Head			
	Val445	Hydrophobic	Core	Leu450	Hydrophobic	Head			
	Pro447	Hydrophobic	Head						
**CXCR3-7D Docked Pose**	**Distance from 7D (2.5 Å)**	**Residue Type**	**7D Region**	**Distance from 7D (3.5 Å)**	**Residue Type**	**7D Region**	**Distance from 7D (5 Å)**	**Residue Type**	**7D Region**
	Leu106	Hydrophobic	Tail	Tyr60	Hydrophobic	Tail	Trp117	Hydrophobic	Core
	Trp109	Hydrophobic	Tail	Ala110	Hydrophobic	Tail	Ala127	Hydrophobic	Core
	Ala113	Hydrophobic	Tail	Asp112	Charged (-Ve)	Tail	Leu190	Hydrophobic	Core
	Phe131	Hydrophobic	HCT	Gly128	Hydrophobic	Core	Gln204	Polar	Core
	Asn132	Polar	Head	Phe135	Hydrophobic	Head	Leu215	Hydrophobic	Head
	**Arg212**	Charged (+Ve)	Head	Asp186	Charged (-Ve)	Head	Gln219	Polar	Head

	Arg216	Charged (+Ve)	Head	His202	Polar	Core	Ser301	Polar	Tail
	Tyr271	Hydrophobic	HCT	Cys203	Hydrophobic	Core	Ser304	Polar	Tail
	Val275	Hydrophobic	Head	**Tyr205**	Hydrophobic	Core	Tyr308	Hydrophobic	Tail
	Ile279	Hydrophobic	Head	Thr213	Polar	Head			
				Trp268	Hydrophobic	Head			
				Tyr271	Hydrophobic	Core			
				His272	Polar	Head			

Table S5: Summary of residues involved in the binding site of 7D for SIRT1 and CXCR3 with interaction types and properties for the minima conformations obtained through free energy landscape plot of both the receptor with 7D.

**Complex Name**	**Residues Involved in Binding Site (5Å)**	**Interaction Type**	**Bond Distance (Å)**	**Type of Residues**
**Sirt1-7D**	**Lys203**	Pi-Cation	3.02	Charged (+Ve)
	Asp204	-	-	Charged (-Ve)
	Leu206	-	-	Hydrophobic
	Pro207	-	-	Hydrophobic
	Glu208	-	-	Charged (-Ve)
	Thr209	-	-	Hydrophobic
	Arg274	-	-	Charged (+Ve)
	Tyr280	-	-	Polar
	Phe297	-	-	Hydrophobic
	Val412	-	-	Hydrophobic
	**Phe413**	H Bond	1.80	Hydrophobic
	**Phe414**	Pi-Pi Stacking	4.64	Hydrophobic
	Gly415	-	-	Hydrophobic
	Glu416	-	-	Charged (-Ve)
	Asn417	-	-	Polar
	**Leu418**	H Bond	1.98	Hydrophobic
	Glu420	-	-	Charged (-Ve)
	Phe422	-	-	Hydrophobic
	His423	-	-	Polar
	Met426	-	-	Hydrophobic
	Pro447	-	-	Hydrophobic
	Leu450	-	-	Hydrophobic
	Ile451	-	-	Hydrophobic
	Ser454	-	-	Polar
**CXCR3-7D**	Arg53	-	-	Charged (+Ve)
	Trp109	-	-	Hydrophobic
	Ala110	-	-	Hydrophobic
	**Asp112**	H Bond	1.83	Charged (-Ve)
	Ala113	-	-	Hydrophobic
	Trp117	-	-	Hydrophobic
	Phe131	-	-	Hydrophobic
	Phe135	-	-	Hydrophobic
	His202	-	-	Polar
	Cys203	-	-	Hydrophobic
	Gln204	-	-	Polar
	**Tyr205**	Pi-Pi stacking	5.09	Hydrophobic
	Phe207	-	-	Hydrophobic
	Arg212	-	-	Charged (+Ve)
	Arg216	-	-	Charged (+Ve)
	Gln129	-	-	Polar
	Leu220	-	-	Hydrophobic
	Trp268	-	-	Hydrophobic
	Tyr271	-	-	Hydrophobic

	His272	-	-	Polar
	Val275	-	-	Polar
	Asp278	-	-	Charged (-Ve)
	Ile279	-	-	Hydrophobic
	Asp297	-	-	Charged (-Ve)
	Lys300	-	-	Charged (+Ve)
	Ser301	-	-	Polar
	Ser304	-	-	Polar
	Tyr308	-	-	Hydrophobic

Table S6: Interaction fingerprint analysis of Sirt1 with 7D and CXCR3 with 7D (minima pose) at different binding site region.

**Sirt1-7D (Minima Pose)**	**Distance from 7D (2.5 Å)**	**Residue Type**	**7D Region**	**Distance from 7D (3.5 Å)**	**Residue Type**	**7D Region**	**Distance from 7D (5 Å)**	**Residue Type**	**7D Region**
	**Lys203**	Charged (+Ve)	Tail	Arg274	Charged (+Ve)	Tail	Asp204	Charged (-Ve)	Tail
	Pro207	Hydrophobic	Core	Tyr280	Hydrophobic	Tail	Leu206	Hydrophobic	Core
	Glu208	Charged (-Ve)	Core	Val412	Hydrophobic	Tail	Thr209	Polar	Core
	**Phe413**	Hydrophobic	Tail	Gly415	Hydrophobic	Tail	Phe297	Hydrophobic	Tail
	**Phe414**	Hydrophobic	Tail	Glu416	Charged (-Ve)	Tail	Ile451	Hydrophobic	Head
	**Leu418**	Hydrophobic	Core	Asn417	Polar	CT	Ser454	Polar	Head
	Glu420	Charged (-Ve)	Head	His423	Polar	Head			
	Phe422	Hydrophobic	Head	Pro447	Hydrophobic	HC			
	Met426	Hydrophobic	Head	Leu450	Hydrophobic	Head			
**CXCR3-7D (Minima Pose)**	**Distance from 7D (2.5 Å)**	**Residue Type**	**7D Region**	**Distance from 7D (3.5 Å)**	**Residue Type**	**7D Region**	**Distance from 7D (5 Å)**	**Residue Type**	**7D Region**
	Trp109	Hydrophobic	Core	Arg53	Charged (+Ve)	Tail	Ala110	Hydrophobic	CT
	**Asp112**	Charged (-Ve)	Core	Ala113	Hydrophobic	Tail	Trp117	Hydrophobic	Core
	Gln219	Polar	Head	Phe131	Hydrophobic	Core	Phe135	Hydrophobic	Head
	Leu220	Hydrophobic	Head	Cys203	Hydrophobic	Core	His202	Polar	Core
	His272	Polar	Head	**Tyr205**	Hydrophobic	Core	Gln204	Polar	Core
	Val275	Hydrophobic	Head	Arg212	Charged (+Ve)	Core	Phe207	Hydrophobic	Head
	Ile279	Hydrophobic	Head	Tyr271	Hydrophobic	HC	Arg216	Charged (+Ve)	Head

	Ser304	Polar	Tail	Lys300	Charged (+Ve)	Tail	Trp268	Hydrophobic	Head
				Ser301	Polar	Tail	Asp278	Hydrophobic	Head
							Asp297	Charged (-Ve)	Tail
							Tyr308	Hydrophobic	CT

Table S7: Interaction analysis during exit path of 7D from Sirt1 and CXCR3 binding pocket.

Complex Name and #Frames	Interacting Residue	Bond Type	Bond Distance (Å)	7D Interacting Region
Sirt1_0-50	Glu410	Aromatic HBond	3.58	Core
Sirt1_51-100	Cys371	Aromatic HBond	3.49	Tail
	Pro399	HBond	1.83	Tail
	Lys408	HBond	1.80	Core
Sirt1_101-150	Cys371	HBond	2.12	Core
	Pro399	HBond	1.91	Tail
Sirt1_151-200	Tyr376	Pi-Pi Stacking	5.39	Head
Sirt1_201-250	Gln421	HBond	2.51	Head
	Lys375	HBond	1.88	Core
Sirt1_251-300	Out from the Pocket			
CXCR3_0-50	Arg212	Aromatic HBond	3.84	Head
	Gln219	Aromatic HBond	3.81	Head
CXCR3_51-100	Arg212	Pi-Cation	5.35	Core
	Arg288	2 Pi-Cation	4.95 and 5.37	Tail
	Lys291	Aromatic HBond	3.10	Tail
CXCR3_101-150	Arg212	2 Pi-Cation	5.30 and 5.65	Core
	Arg288	Pi-Cation	5.20	Core
CXCR3_151-200	Pro208	Aromatic HBond	3.79	Head
	Arg212	2 Pi-Cation	5.02 and 5.17	Core
	Arg288	Pi-Cation	4.24	Core
CXCR3_201-250	Arg197	2 Pi-Cation	5.99 and 6.16	HC
	Asp278	Aromatic HBond	3.33	Head
CXCR3_251-300	Out from the Pocket			

Table S8: Summary of representative active, weak, and inactive analogues of 7D reported in our previous studies together with their biochemical/cellular activity profiles (in-vitro data). In the table **(A)** we integrate previously reported Sirt1 inhibition data with docking, induced-fit docking, MM/GBSA and experimental % inhibition, and **(B)** CXCR3 computational analyses to provide structural interpretation of the observed SAR trends and proposed dual-target interaction behaviour. Only most active analogues were used in CXCR3.

A

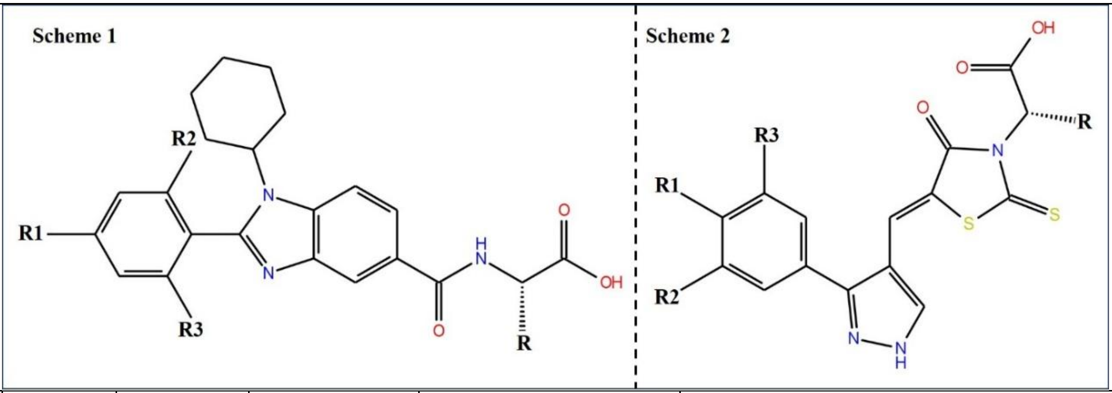

Scheme 1 Comp.	R	R1	R2/R3	Mol. Mass	Static Docking (kcal/mol^{-1})	IF Guided Docking Sirt1		% Inhibition at 10µM Sirt1
						Dock Score ΔG Bind (kcal/mol^{-1})	ΔG Bind (kcal/mol^{-1})	
7a	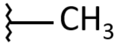	-O*Me*	-H	421	-9.23	-6.26	-58.96	88.03
7b	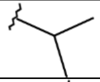	-O*Me*	-H	450	-7.98	-7.06	-59.49	88.18
7c	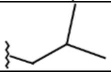	-O*Me*	-H	464	-9.15	-6.68	-58.85	86.34
7d	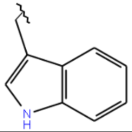	-O*Me*	-H	537	-8.81	-6.70	-72.08	89.99
7e	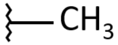	-F	-H	409	-11.47	-6.51	-54.45	<30
7f	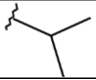	-F	-H	437	-9.32	-7.40	-54.66	<30

7g	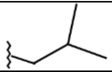	-F	-H	451	-8.76	-6.08	-58.20	80.82
7h	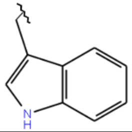	-F	-H	525	-8.67	-5.84	-61.60	86.83
7i	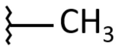	-H	-Cl/F	472	-10.70	-7.08	-49.04	<30
7j	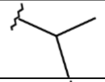	-H	-Cl/F	486	-9.71	-5.33	-33.49	<30
7k	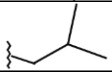	-H	-Cl/F	559	-8.49	-5.61	-52.97	<30
7l	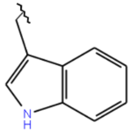	-H	-Cl/F	559	-8.12	-6.61	-56.97	<30
**Scheme 2 Comp.**								
13d	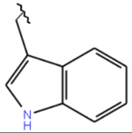	-O*Me*	-H	504.58	-6.90	-7.26	-63.40	90.64
13h	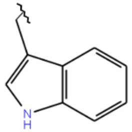	-H	-F	510.53	-7.88	-7.21	-60.23	89.15
13l	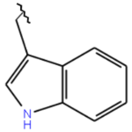	-NO_2	-H	519.55	-5.50	-7.09	-70.88	89.64
**Control**		**Ex527**				-7.78	-63.58	97.73
**Activity on CXCR3**								
7d	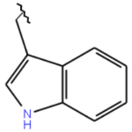	-O*Me*	-H	537	-8.06		-62.58	NA

13d	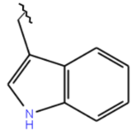	-*OMe*	-H	504.58	-9.27		-62.12	NA
13h	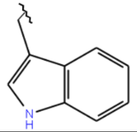	-H	-F	510.53	-8.93		-59.50	NA
13l	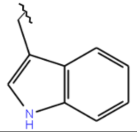	-*NO*$_2$	-H	519.55	-10.46		-63.01	NA
**Control**		**AMG487**				-5.57	-66.37	NA

B

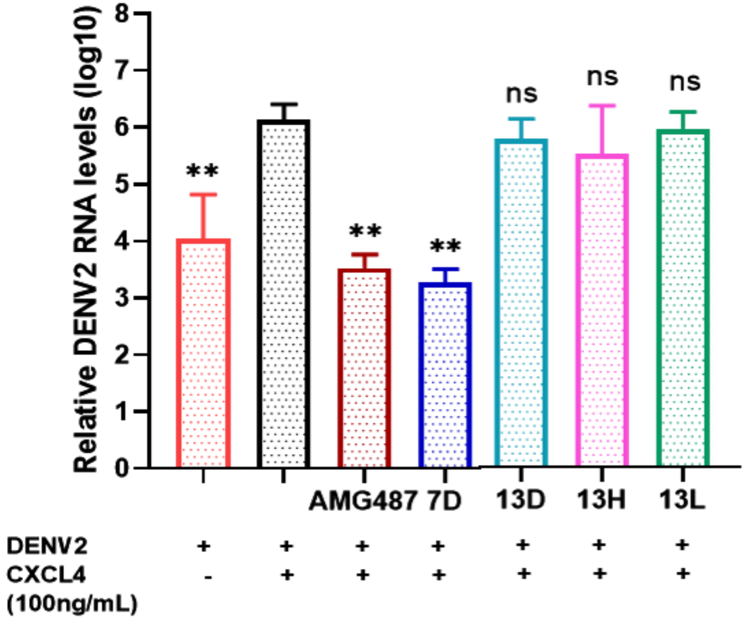

Supplement: Document S1. Figures S1–S11 and Tables S1–S8 [file mmc1.pdf]
